# Distinct epigenetic features of differentiation-regulated replication origins

**DOI:** 10.1186/s13072-016-0067-3

**Published:** 2016-05-10

**Authors:** Owen K. Smith, RyanGuk Kim, Haiqing Fu, Melvenia M. Martin, Chii Mei Lin, Koichi Utani, Ya Zhang, Anna B. Marks, Marc Lalande, Stormy Chamberlain, Maxwell W. Libbrecht, Eric E. Bouhassira, Michael C. Ryan, William S. Noble, Mirit I. Aladjem

**Affiliations:** DNA Replication Group, Developmental Therapeutics Branch, Center for Cancer Research, National Cancer Institute, National Institutes of Health, Bethesda, MD 20892 USA; In Silico Solutions, Falls Church, VA 22033 USA; Department of Genetics and Developmental Biology, University of Connecticut Health Center, Farmington, CT 06032 USA; Department of Computer Science and Engineering, University of Washington, Seattle, WA 98195 USA; Department of Cell Biology, Albert Einstein College of Medicine, Bronx, NY 10461 USA; Department of Genome Sciences, University of Washington, Seattle, WA 98195 USA

**Keywords:** Origin of replication, Chromatin, Histone modification, Cellular differentiation, CpG islands, H3K4me3, H3K9Ac, H3K9me3, Cell cycle, Proliferation

## Abstract

**Background:**

Eukaryotic genome duplication starts at discrete sequences (replication origins) that coordinate cell cycle progression, ensure genomic stability and modulate gene expression. Origins share some sequence features, but their activity also responds to changes in transcription and cellular differentiation status.

**Results:**

To identify chromatin states and histone modifications that locally mark replication origins, we profiled origin distributions in eight human cell lines representing embryonic and differentiated cell types. Consistent with a role of chromatin structure in determining origin activity, we found that cancer and non-cancer cells of similar lineages exhibited highly similar replication origin distributions. Surprisingly, our study revealed that DNase hypersensitivity, which often correlates with early replication at large-scale chromatin domains, did not emerge as a strong local determinant of origin activity. Instead, we found that two distinct sets of chromatin modifications exhibited strong local associations with two discrete groups of replication origins. The first origin group consisted of about 40,000 regions that actively initiated replication in all cell types and preferentially colocalized with unmethylated CpGs and with the euchromatin markers, H3K4me3 and H3K9Ac. The second group included origins that were consistently active in cells of a single type or lineage and preferentially colocalized with the heterochromatin marker, H3K9me3. Shared origins replicated throughout the S-phase of the cell cycle, whereas cell-type-specific origins preferentially replicated during late S-phase.

**Conclusions:**

These observations are in line with the hypothesis that differentiation-associated changes in chromatin and gene expression affect the activation of specific replication origins.

**Electronic supplementary material:**

The online version of this article (doi:10.1186/s13072-016-0067-3) contains supplementary material, which is available to authorized users.

## Background

Proliferating eukaryotic cells duplicate their genomes exactly once each cell division cycle with remarkable fidelity, ensuring that all genetic and epigenetic information is accurately transferred to daughter cells. In most somatic metazoan cells, chromosome replication starts at numerous, consistent initiation sites (“replication origins”) and advances in a precise temporal- and tissue-specific order [1–3]. Uncoordinated, incomplete or excessive replication can cause genomic instability, which can lead to developmental abnormalities and cancer. Consistent with a role in coordinating replication with gene expression, individual replication origins can modulate chromatin structure to affect transgene expression in vectors used for cellular reprogramming [3–6]. Despite their essential role, metazoan replication origins do not share an obvious, stringent consensus sequence, unlike those identified in bacteria and yeast [2, 7–11]. Instead, metazoan origins tend to contain flexibly defined common sequence motifs, such as A/T or G/C skews, transcription factor-binding motifs [12, 13], CpG islands [9, 14, 15], G-quadruplexes [7] and sequence asymmetry [11, 16]. This sequence versatility suggests that primary DNA sequences are not the sole determinants of replication initiation events, and origin activity might depend on both genetic and epigenetic features.

The steps that lead to replication initiation in eukaryotes involve highly conserved DNA–protein interaction cascades. Replication initiation requires the recruitment of pre-replication complexes that nucleate on the origin recognition complex (ORC) [1, 17–21] and the mini chromosome maintenance complex (MCM) helicase. Pre-replication complexes are inactive when loaded onto chromatin; their activation requires the recruitment of additional proteins to form the CMG (Cdc45, MCM and GINS) complex [22]. Proteins that are essential for replication (such as ORCs) exhibit DNA sequence-specific binding to replication origins in budding yeast but not in metazoans, consistent with the lack of a consensus sequence for the initiation of metazoan DNA replication [23, 24]. Notably, pre-replication complexes within each cell are more numerous than actual replication initiation sites, and only a fraction of potential replication origins initiate replication during each cell cycle [2, 3, 25].

Because mammalian replication origins do not share a clear consensus sequence, the mechanisms that dictate the choice of replication origins in mammalian systems have been difficult to decipher [1, 2]. Use of all potential replication initiation sites is not strictly required for DNA replication, but their presence is necessary for genomic stability [3, 26], and a recent simulation study showed that the locations of replication origins (the initiation probability landscape) could predict the distribution of replication timing domains [27]. Hence, the observed consistency of replication origins might be necessary to determine the time of replication and to coordinate DNA synthesis with other chromatin transactions such as transcription, DNA repair and chromosome condensation. Epigenetic regulation of DNA replication may allow transcription and replication to proceed in a coordinated manner, consistent with the existence of tissue-specific replication origins.

Several lines of evidence suggest that chromatin modifications play a role in coordinating replication and transcription. First, maps delineating the locations of replication initiation events, which can be created using nascent strand preparations combined with whole-genome mapping approaches such as next-generation sequencing [9], suggest that metazoan initiation sites share some chromatin modifications [28–32]. Although no particular histone modification examined thus far has exhibited a striking functional association with all replication origins, certain sequence elements and histone modifications, like methylation on histone H3 Lysine 79, have been associated with replication [33]. Second, functional studies [34–38] revealed that replication initiation sites contain sequence elements (replicators) that are genetically required to start replication, but robust similarities among such sequences are not evident. Replicator sequences can affect chromatin structure, as demonstrated by their ability to prevent transcriptional silencing [4] by facilitating distal interactions involving a chromatin remodeling complex [39]. Third, distal DNA elements, which do not start replication but facilitate chromatin remodeling, interact with replicators and are required for replication initiation at several loci (e.g., human beta-globin (*HBB*) [40], Chinese hamster *Dhfr* [41] and murine *Th2* [42]). Lastly, replication initiation events are enriched in moderately transcribed genomic regions and are depleted in regions that are not transcribed or that exhibit very high rates of transcription [9]. These observations support the notion that initiation of DNA replication from potential replication origins is a dynamic process that can affect, and be affected by, chromatin transactions.

Cellular differentiation influences replication timing over large genomic regions (400–800 kb), and chromatin domains that replicate concomitantly are often located in distinct nuclear compartments in human and mouse cells [43]. The distribution of replication timing domains, which can be predicted in simulation studies by the locations of replication origins [27], dynamically responds to differentiation cues and closely reflects the spatial organization of chromatin [30, 31]. Changes in replication timing sometimes, but not always, reflect changes in gene expression [44]. In general, early replicating regions are gene rich, show no correlation with gene expression and contain both active and inactive genes. Late replicating regions are generally gene poor and contain mostly silent genes, and their replication timing is often correlated with differentiation-induced gene expression activation [30].

Here, we tested whether cellular replication origin subsets shared specific DNA and chromatin modifications. We specifically searched for chromatin modifications preferentially associated with replication origin sequences as compared to flanking sequences. Since cells of divergent lineages differed in the locations of replication initiation events [7, 9], we investigated whether cell-type-specific origins and shared origins were associated with distinct chromatin modifications.

## Methods

### Nascent strand preparation

We performed nascent strand DNA preparation using two methods: λ-exonuclease digestion of DNA fragments that lack an RNA primer and bromodeoxyuridine (BrdU) labeling of replicating DNA [45]. For the λ-exonuclease digestion, DNA was extracted from asynchronous cells and was fractionated on a neutral sucrose gradient. Fractions of 0.5–2.5 kb were treated with λ-exonuclease to remove non-RNA-primed genomic fragments. For the BrdU-labeling method, asynchronously growing cells were incubated with BrdU for 20 min. DNA was extracted and size fractionated. Short, BrdU-labeled DNA, which corresponded to origin-proximal newly replicated fragments, was isolated by immunoprecipitation using antibodies targeted against BrdU-substituted DNA. Pooled nascent strand libraries prepared with both methods were sequenced using paired-end 101-bp reads with TruSeq V3 chemistry on a Hiseq 2000 sequencing system. Samples were trimmed of adapters using Trimmomatic Software and aligned to the human genome (hg19) using Burrows–Wheeler Aligner (BWA) software.

### Calling replication origin peaks

Following sequencing, peaks identifying genomic regions enriched in nascent strand reads were called by comparing BAM files containing the aligned nascent strand DNA sequences to BAM files containing control, sonicated genomic DNA sequences. To control for copy number variations that are prevalent in cancer cells, each nascent strand BAM file was compared to a corresponding BAM file containing genomic DNA sequences from the same cell line (for a list of cell lines see Additional file 1: Table S1a).

For peak calling, we used the SICER program, which was designed to identify broad peaks from chromatin immunoprecipitation [ChIP]-seq experiments against histone modifications and is efficient at identifying replication origins [47]. SICER parameters were as follows: redundancy threshold = 2, window size = 200, fragment size = 150, gap size = 600, FDR = 0.01, p value = 0.05. SICER outputs a list of peak locations and sizes in a BED (Browser Extensible Data)-formatted file that was used for further analyses. To test whether the DNA preparations indeed corresponded to regions that included replication origins, we visualized sequencing data at well-characterized replication origin sites (DHFR, beta-globin, DBF4; Additional file 1: Fig. S1a–c) on a genome browser in parallel with using real-time PCR to analyze replication initiation.

To control for method-specific biases in nascent strands obtained with λ-exonuclease digestion, we also called peaks from K562 and MCF7 nascent strands isolated by λ-exonuclease digestion against BAM files aligning λ-exonuclease-digested genomic DNA reads from K562 G1 cells and MCF7 G0 cells [46], respectively. K562 λ-exonuclease-digested genomic DNA was prepared from elutriated K562 cells; reads from MCF7 G0 λ-exonuclease-digested genomic DNA were obtained from SRA045284. We also used genomic regions that exhibited λ-exonuclease digestion biases in both K562 and MCF7 cells to control for λ-exonuclease digestion biases in nascent strand preparations obtained from U2OS and iPS cell lines, for which λ-exonuclease-digested G0 DNA was not available ([46]; see “BED file intersections and subtractions” section). Peak files corrected against λ-exonuclease digestion biases exhibited above 90 % similarity to peaks called against undigested sonicated genomic DNA (see Additional file 1: Table S1b for an example using MCF7 origin data) and contained fewer CpG islands (2 % fewer CpG islands in K562 cells and 10 % fewer CpG islands in MCF7 cells) as expected given the high abundance of CpGs in λ-exonuclease-digested DNA [46].

To control for method-specific biases in nascent strands obtained with the BrdU-labeling and immunoprecipitation methods, we also called peaks from BAM files representing nascent BrdU-substituted DNA against BAM files representing DNA sequences from a preparation of sonicated, uniformly BrdU-substituted DNA originating from an asynchronous culture grown in the presence of BrdU for 48 h. Peaks called against BrdU-substituted DNA exhibited >95 % similarity with peaks called against unsubstituted sonicated genomic DNA (see Additional file 1: Table S1b for an example using HCT116 data).

### BED file intersections and subtractions

BED file intersections and subtractions were performed using a custom script (available upon request). The script accepts two BED files as input and designates one file as a “reference” and the other as a “comparator.” The intersection script produces a BED file that lists peaks from the reference file that overlap within 2 kb of peaks in the comparator file. The subtraction file lists peaks from the reference file that do not overlap within 2 kb of peaks in the comparator file. Outputs therefore differ depending on the identity of the file that was designated as the reference and contain only reference file peaks. Intersections were performed to identify peaks shared among several cell lines. These peaks correspond to the locations of shared replication origins. Similarly, subtractions were performed to identify cell-type-specific origins.

We used BED file subtractions and intersections to correct computationally for λ-exonuclease digestion biases in nascent cell preparations. We first created two BED files for each MCF7 and K562 cells: The first file contained nascent strand peaks called against genomic DNA and the second contained nascent strand peaks from the same cell line called against λ-exonuclease-digested DNA. As reported previously [46], the latter files contained a subset of the peaks present in the former file. We then used the BED file subtraction scripts to identify peaks, for each cell line, that were present in the first file and not in the second file (λ-exonuclease-bias-generated peaks): genomic regions that were resistant to λ-exonuclease digestion but were not further enriched in newly replicated RNA-primed DNA. We then used the file intersection script to create a BED file that contained λ-exonuclease-bias-generated peaks appearing in both cell lines (this step further enriched for λ-exonuclease-bias-generated peaks, which reflect the primary DNA sequences and are therefore expected to appear in all cells regardless of replication status and epigenetic modifications). This file was subtracted from nascent strand peak files called against genomic DNA from U2OS and iPS cells.

### Colocalization analyses

Colocalization analyses comparing the locations of replication origins with genetic features and chromatin modifications were performed using the Web-based ColoWeb program (http://projects.insilico.us.com/ColoWeb/) and the Genomatix suite (https://www.genomatix.de/). We quantified the abundance of chromatin modifications (DNase-hypersensitive sites, covalent histone modifications and CpG islands) within 20 kb of replication origins for each cell line using known chromatin modifications from the same cell line that has been deposited in public datasets and preloaded into ColoWeb [48]. We used known chromatin modifications from K562 and H1ES cells to assess colocalization with replication origins from cells of similar differentiation status. Known chromatin modifications from K562 cells were used to analyze erythroid cells (K562 cells and basophilic erythroblasts (EB) primary cells). Similarly, known chromatin modifications from H1ES cells were used to analyze pluripotent H1ES (embryonic stem), AS_iPS (induced pluripotent) and PWS_iPS (induced pluripotent) cell lines.

The ColoWeb analysis produced a shaded scatterplot graphically summarizing the locations and densities of chromatin features relative to each origin region. ColoWeb also calculated the general background density of each chromatin feature and created a histogram denoting the local distribution of each chromatin modification. For each chromatin feature, the above-mean-integral (AMI) value corresponded to the frequency of that particular feature near replication origins exceeding the general background in flanking regions. AMIs reflecting colocalization between origins and chromatin modifications, CpG methylation and DNase hypersensitivity were calculated for each cell line. Origins from HCT116 and U2OS cells were used to identify shared origins, but could not be used directly in chromatin analyses because chromatin data for these cell lines are scarce in public databases.

ColoWeb was also used to measure the abundance of nascent strands in 20-kb regions centered on each chromatin feature (feature-centered analysis). Feature-centered analyses and replication origin-centered analyses produced highly similar results for all chromatin features tested.

### Cluster generation and replication timing analyses

ColoWeb analyses were performed using BED files containing all replication origin peaks from each cell line, as well as BED files resulting from intersections and subtractions for shared and cell-type-specific replication origins, respectively. These analyses produced AMI values quantifying the extent of colocalization of replication origins with chromatin modifications. Tab-delimited files containing mean-centered AMI values were clustered using CIMminer [49]. The “correlation” distance algorithm was used for clustering, and the “equal width” binning algorithm assigned colors to values.

For replication timing analyses, K562 cell origins were stratified by intersecting replication origin BED files with replication timing files as recently described [11]. Replication origin colocalization with selected histone modifications was assessed using the Genomatix suite. Additionally, the semiautomated genome annotation (SAGA) algorithm was used to determine origin distribution and abundance in each timing group within the following chromatin domains: BRD: “broad expression domain,” genes that are broadly expressed across cell types; CON: “constitutive heterochromatin,” permanently silent regions; FAC: “facultative heterochromatin,” genes specific to a cell type other than K562; QUI: “quiescent,” lacking any activity; SPC: “specific expression domain,” genes expressed in K562 cells, but not many others.

## Results

### Shared and cell-type-specific replication origins

We created a comprehensive dataset of human replication origins to assess differentiation- and cancer-related variations in origin usage and to identify chromatin modifications that locally distinguish replication origins. We analyzed replication origin data from eight cell lines, combining previously mapped data (Additional file 1: Table S1a; [9, 50–52]) with new data (accession number: GSE80391) from U2OS osteosarcoma cells and two iPS cell lines, AS_iPS and PWS_iPS [53].

We sequenced nascent strands (NS-Seq) collected from asynchronous human cells by two methods [45]: short, λ-exonuclease-resistant DNA fragments and short, BrdU-substituted DNA fragments. These two isolation methods rely on non-overlapping assumptions [45] and were used to minimize method-specific biases [46]. Replication origin peaks identified by both methods had average widths of 3–5 kb, and the number of replication origins identified in the cell lines studied varied from ~80,000 to ~200,000 (Additional file 1: Table S1a). The number of origins and their distributions among genic and non-genic regions (Additional file 1: Table S1c) were in agreement with prior studies [7, 9, 10, 51, 54]. Similar to previous studies, replicates exhibited high reproducibility, measured as the agreement between biological replicates [9, 50] and by the consensus among nascent strands isolated by λ-exonuclease resistance and by BrdU substitution ([51]; Additional file 1: Table S1b). High concordance (84.5 % of peaks) was also observed when we compared our K562 nascent strands preparation with an independent K562 nascent strand preparation despite using a different peak calling method [54].

To determine whether cells of the same differentiation state from two unrelated genetic backgrounds would activate similar replication origins, we mapped origins in two independently derived iPS cell lines, AS_iPS and PWS_iPS. We evaluated the proportion of origin peaks that were located within 2 kb of each other in these two samples. As shown in Additional file 1: Table S2a, 87.9 % of the origins in AS_iPS cells localized within 2 kb of origins in PWS_iPS cells, whereas 59.1 % of origin peaks with h1ES cells exhibited similar colocalization (Additional file 1: Table S2a, compare row 1 with row 2). Only 56.5 % of origin peaks were present in all iPS, H1ES and EB cells (Additional file 1: Table S2a, row 4), suggesting that the locations of some replication origins might be affected by differentiation state. Similarly, 32.2 % of replication origins were present in all four cancer cell lines used in the study (Additional file 1: Table S2b, row 5; see Additional file 1: Fig. S1a–c for examples of colocalization among origins in different cell lines).

**Fig. 1:**
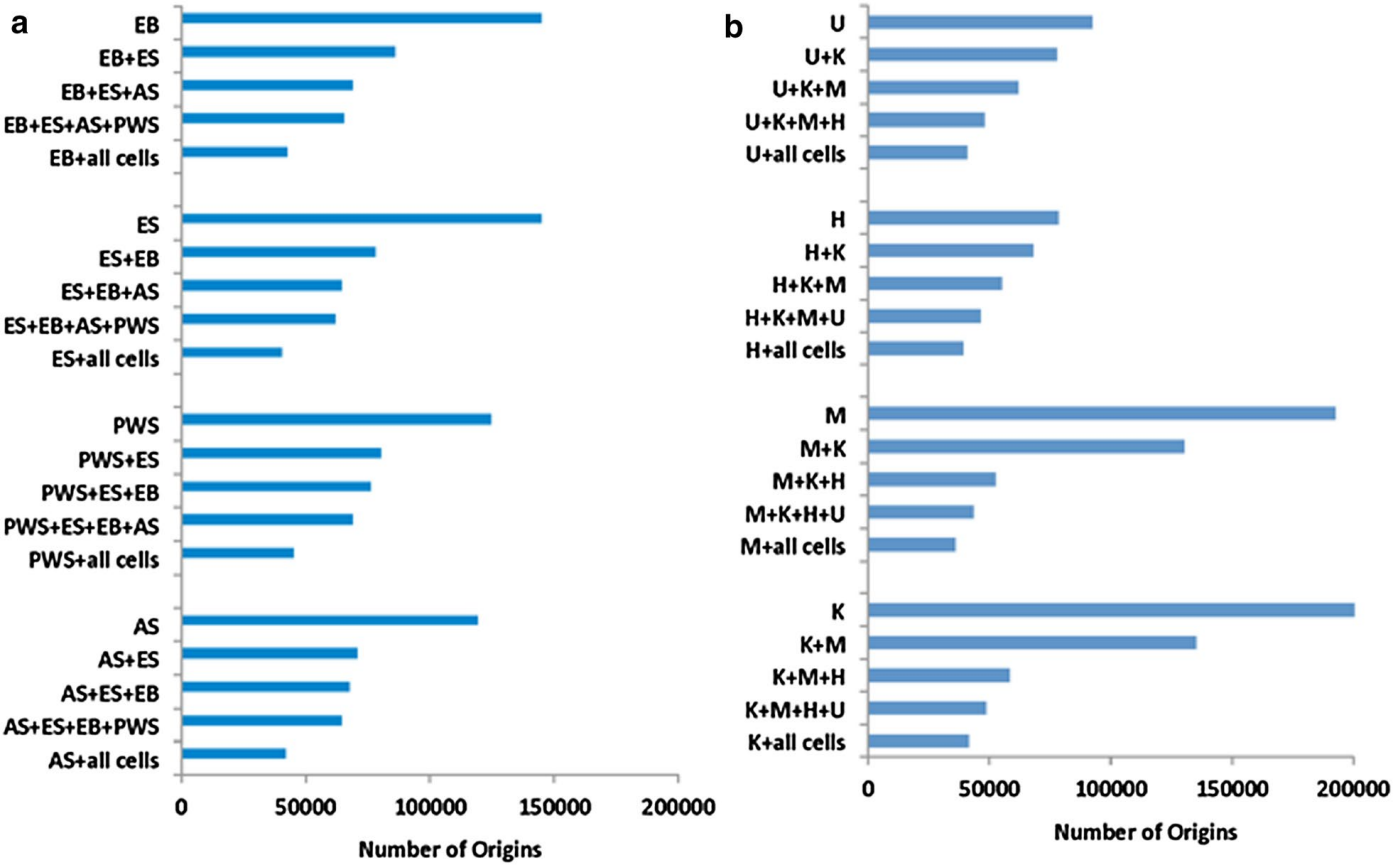
A recurrent group of shared replication origins in normal and cancer cells. The numbers of shared replication origins among **a** normal and **b** cancer cells. For each cell line, the overall number of origin peaks is plotted in the *top column* followed by the number of origins in that cell line that were also present in the other cells indicated (sequential intersections; for details, see “Methods” section and Table 1 and Additional file 1: S2a–c). For example, in panel a, top group, EB represents the number of origin peaks present in the EB sample; EB + ES depicts the number of origin peaks present in the EB sample that were also present in the ES sample; EB + ES + AS depicts the number of origin peaks present in the EB sample that were also present in the ES and the AS samples, etc. The *last column* for each cell line group shows the number of origins remaining following sequential intersections with **a** all four normal cell lines or **b** all four cancer cell lines. Normal cell lines were AS (AS_IPS), PWS (PWS_IPS), ES (H1ES) and EB. Cancer cell lines were K562 (K), MCF7 (M), HCT116 (H) and U2OS (U)

 For further analyses, we have characterized two sets of origins, shared and cell specific, for each cell line. We defined “cell-specific” origins as replication origins that were found only in the indicated cell line and did not colocalize (no peaks located within 2 kb) with origin peaks in any of the other cell lines in the cohort (the cancer cell cohort included K562, MCF7, U2OS and HCT116 cell lines, and the non-cancer cell cohort included ES, EB and both iPS cell lines). We defined “shared origins” as replication origins that were present in the indicated cell line and colocalized (peaks located within 2 kb) with origin peaks found in all other cells within the cohort. Although the fraction of shared origins in each cell line varied, the number of shared origins was similar in cancer and non-cancer cell lines and a set of 36–45,033 origins was present in all eight cell lines (Fig. 1). As shown in Fig. 1, origins that were present in a pair of cell lines were likely to be shared among additional cells. Shared origins were more likely to localize to promoters, whereas cell-type-specific origins were more prevalent in intergenic regions (Additional file 1: Table S1C).

Because cell-type-specific origins appeared in only in a few samples, we performed an additional test to determine whether or not those cell-type-specific origins indeed represented reproducible replication origins. We used the irreproducible discovery rate (IDR) analysis [55], designed to quantify the reproducibility of biological replicates, as a tool to assess the reproducibility of shared and cell-type-specific nascent strand peaks. IDR creates a curve that quantitatively assesses data point consistency across replicates, and then calculates a reproducibility score based on the fraction of data points that deviate from the curve. We compared the reproducibility scores of shared and cell-type-specific replication origins from AS_IPS and PWS_IPS cells and, separately, from AS_IPS and U2OS cells (Additional file 1: Fig. S2a, b). Shared and cell-type-specific origins from the AS_IPS and PWS_IPS lines had similar reproducibility scores, but this was not observed when we compared AS_IPS and U2OS cells. These analyses suggested that cell-type-specific origins, although limited to a few of the cell types tested in our analyses, reflected consistent and reproducible initiation events.

### Chromatin modifications associated with distinct groups of replication origins

**Fig. 2:**
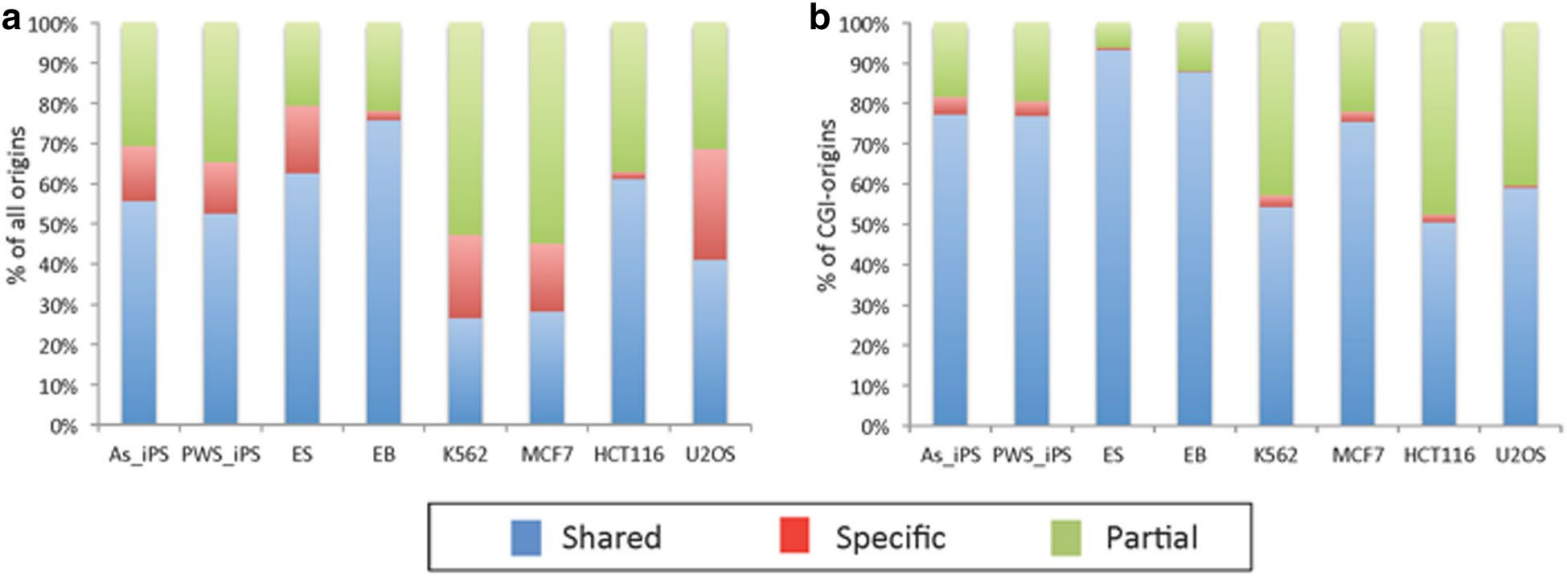
CpG islands (CGIs) are significantly enriched among shared replication origins. Distribution of **a** all replication origins in the indicated cells and **b** origins associated with CpG islands (CGI origins). Origins were stratified as shared and cell type specific (for a definition of shared and cell-type-specific origins, see the text and legend to Table 1) or partially shared (origins initiating replication in some cells, but not others). Distributions are displayed in 100 % stacked column charts

Previous studies suggested that mammalian replication origins associate with CpG islands (CGIs) [9, 14, 15]. We asked whether CpG islands associated with shared or cell-type-specific origins. We found that a large majority (75–96 %) of all CpG islands associated with replication origins. Notably, since there are more origins than CpG islands overall, only 7–25 % of origins associated with CpG islands (Table 2). Ori-CGIs in both normal and cancer cells were more commonly associated with shared origins than with than cell-type-specific origins (Fig. 2; Table 2, p < 2.2 × 10^−16^ for all samples except HCT116, p < 0.001).

**Table 1:** Characterization of replication origins in cancer and non-cancer cells

Row #	Cell line	# of All origins	# Shared origins	# of Cell-specific origins	% Shared	% Cell specific
1	ES	90,621	62,477	8507	68.9	9.4
2	EB	144,753	65,868	18,366	45.5	12.7
3	AS_iPS	119,944	67,977	14,896	56.7	12.4
4	PWS_iPS	135,397	76,642	16,471	56.6	12.2
5	K562	202,653	64,972	35,266	32.1	17.4
6	MCF7	193,118	62,930	26,100	32.6	13.5
7	HCT116	78,859	57,873	1319	73.4	1.7
8	U2OS	92,814	60,631	3955	65.3	4.3

Replication origins were identified as regions enriched in nascent strand reads in four non-cancer cell lines (ES, EB, AS_iPS and PWS_iPS) and in four cancer cell lines (K562, MCF7, HCT116, U2OS). The number of replication origins is the number of origin peaks called versus the appropriate genomic control for each cell line (see “Methods” section and Additional file 1 for details and reproducibility data). The number of shared origins is the number of origin peaks that were present in the indicated cell line and in all other cells of the same group (non-cancer cells for ES, EB and the two iPS lines and cancer cells for K562, MCF7, HCT116 and U2OS). The number of cell-specific origins is the number of origin peaks that were present in the reference cell line, but not in any of the other cells of the same group. The “% shared” peaks and the “% cell-specific” peaks represent the percentage of shared and cell-specific peaks, respectively, versus the overall number of peaks in the indicated file

**Table 2:** Percentage of CGIs that are replication origins and percentage of origins that are CGIs

Cell line	% CGIs that are origins	% Origins that are CGIs
AS_IPS	96.06	19.00
PWS_IPS	96.64	17.11
ES	75.60	20.01
EB	92.34	22.69
K562	80.69	10.84
MCF7	85.80	7.08
HCT116	88.53	24.97
U2OS	75.78	15.32

**Fig. 3:**
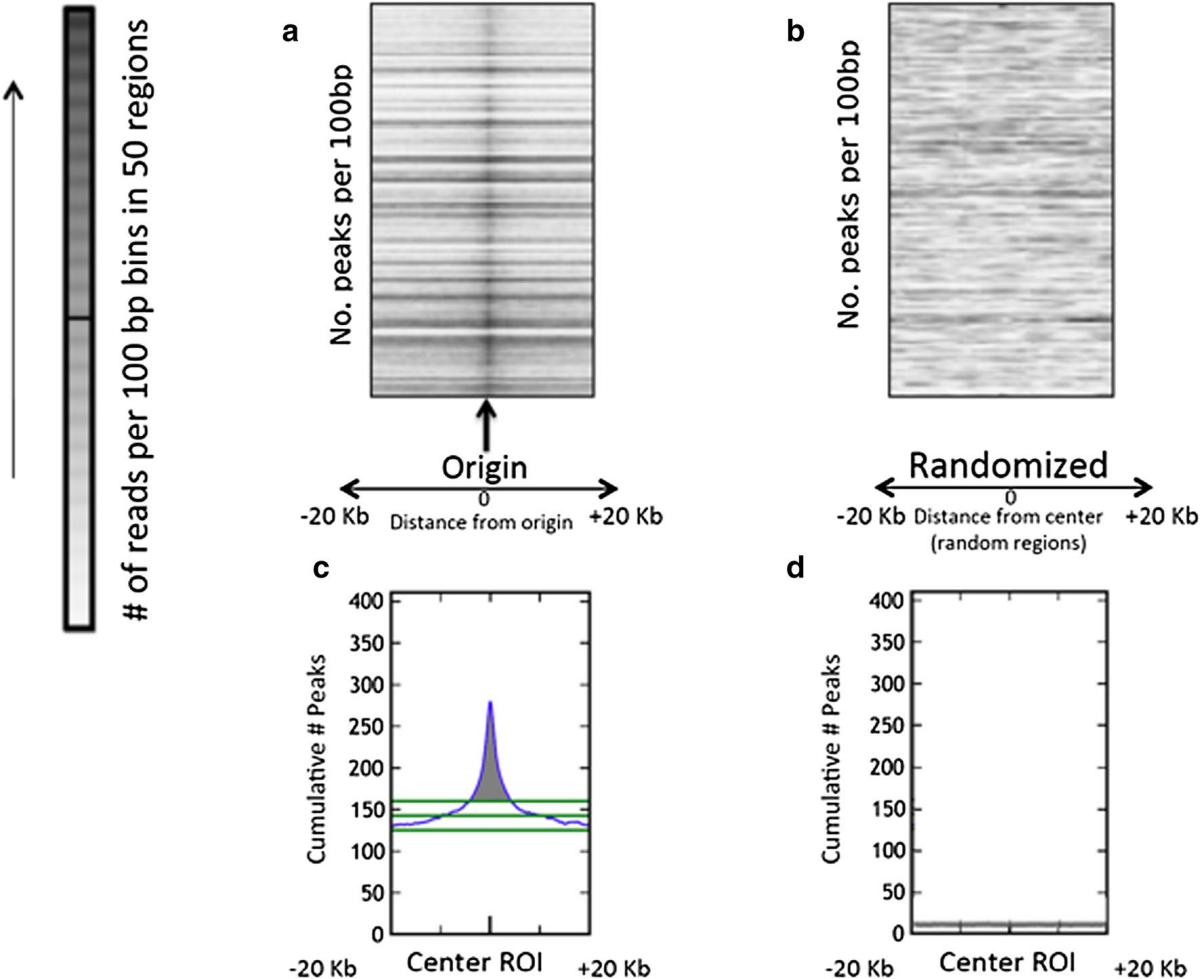
Example ColoWeb output: comparison of the distribution of K562 replication origins to K562 histone modification H3K4me3. The *x* axis represents distance from the center of **a** replication origins or **b** randomized regions. Each scatterplot contains 100 rows. *Each row* contains data for 50 randomly selected regions [origin-containing regions in (**a**) and randomized fragments of the same GC content in (**b**)], divided across 100 bases bins. The *grayscale* corresponds to the extent of H3K4 trimethylation in each bin. **c**, **d** Graphs summarizing the colocalized peaks for the analyses represented in (**a**, **b**), respectively. The *green horizontal lines* for the mean and high/low oscillation values (40th and 60th percentiles, respectively) are shown on the histogram. The *shaded area*, covering the region under the peak and above the upper variance level [48], corresponds to the above mean integral (AMI) used in colocalization studies. For more examples of scatterplots, see Additional file 1: Fig. S3

We next asked whether local CpG methylation and other chromatin modifications preferentially associated with shared and cell-type-specific origins. We quantified the extent of preferential origin using the Web-based tool ColoWeb (for details, see “Methods” section) (http://projects.insilico.us.com/ColoWeb/index.jsp [48]). ColoWeb was designed to identify modifications that exhibited higher association with replication origins than with adjacent sequences because we were interested in chromatin modifications that locally marked replication initiation events. Using ColoWeb, we created a dataset of all 20-kb genomic fragments flanking replication origins and then mapped the distribution of chromatin modifications within those fragments. For example, as shown in Fig. 3, H3K4-trimethylated regions exhibited markedly high intensity at replication origins (the center of the scatterplot; Fig. 3a) and produced a clear origin-centered peak on the accompanying histogram (Fig. 3c). A similar distribution was not observed when the same trimethylated H3K4 sites were aligned to a randomized file containing genomic regions not enriched for replication origins (Fig. 3b, d).

**Fig. 4:**
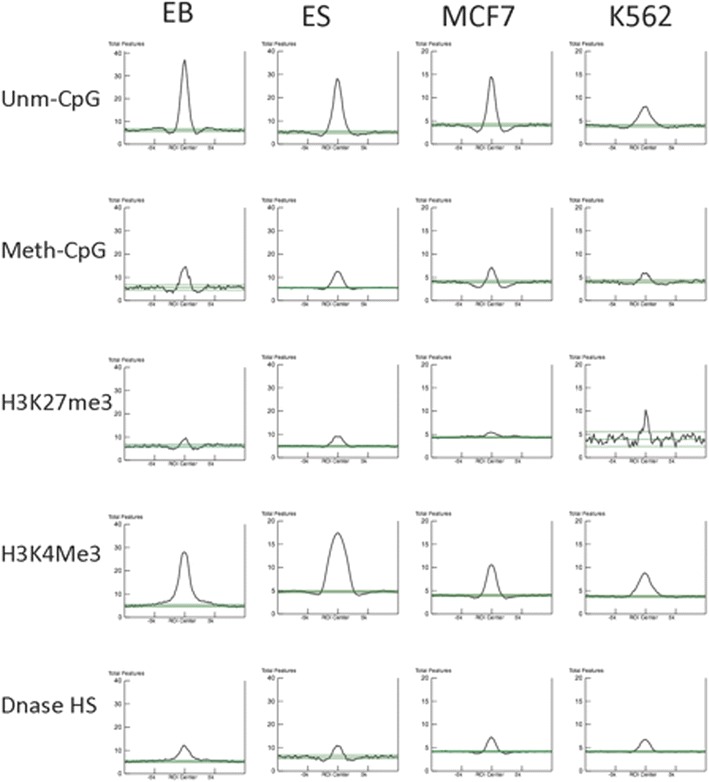
Association of replication origins with chromatin features. Representative ColoWeb alignments of chromatin features with replication origins from several cancer and non-cancer cell lines. Only cell lines that were extensively characterized for chromatin modifications in the literature (ES, MCF7 and K562, with EB origins analyzed vs. K562 modifications) were included in this analysis. AMI values corresponding to the histograms are shown in Additional file 1: Table S4 and scatterplots are shown in Additional file 1: Fig. S3

We used ColoWeb to quantify colocalization between origins and various histone modifications by measuring above-background histogram values (Above Median Integrals, or AMIs, representing the integral of areas above the background level and under the peaks; for an example, see the shaded area in Fig. 3c). We then used AMI values to provide an overview of the association of replication origins with chromatin modifications in all cell lines for which chromatin modification data were available. For example, as shown the top row of Fig. 4, unmethylated CpGs (Unm-CpG) exhibited strong preferential colocalization with origin peaks, whereas methylated CpG (Meth-CpG) exhibited a lower level of colocalization (Fig. 4, second row). These strong association with unmethylated CpGs and weaker association with methylated CpG were reflected in the AMI values reported in Additional file 1: Table S4. Similarly, trimethylation of H3K27 exhibited only minor preferential association with origins (Fig. 4 third row), whereas trimethylation of H3K4 was preferentially associated with replication origins when compared with adjacent sequences (Fig. 4 row 4 and Additional file 1: Table S4). Surprisingly, although replication origins are known to be abundant in regions that exhibit DNase hypersensitivity, DNase-hypersensitive sites did not associate preferentially with replication origins when compared with adjacent regions (Fig. 4 row 5). RNA polymerase II binding sites and trimethylation of histone H3K9 also showed moderate association with replication origins in most cell lines (Additional file 1: Table S4).

**Fig. 5:**
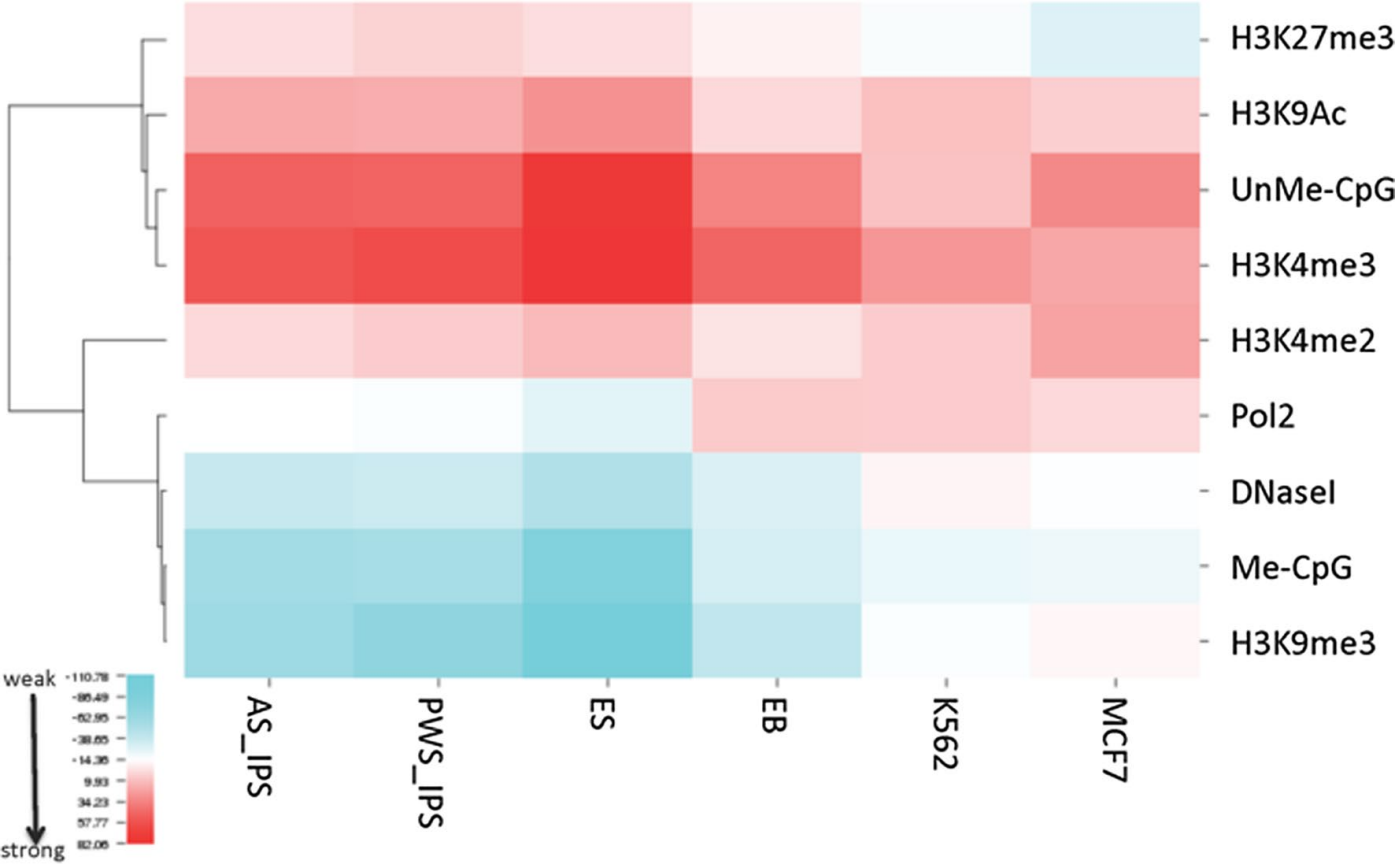
Replication origins clustered by preferential association with chromatin features. A heat map showing clustered standardized mean-centered AMI values (for examples, see Fig. 4, Additional file 1: Fig. S3 and Additional file 1: Table S4) representing the extent of preferential association between origins and chromatin markers. For each chromatin modification, AMI values measure the extent of association with replication origins exceeding the general association of the same modification with flanking regions. The map, clustered by both cell line and chromatin feature, is *color coded*, with *deep red* representing higher mean-centered AMI values and *deep blue* representing lower values (origins from the cancer cell lines U2OS and HCT116 cells were not included in this clustered analysis due to the scarcity of available chromatin data). Replication origins associated strongly with unmethylated CpGs and H3K4me3 and, to a lesser extent, with H3K9 acetylation

We collected AMI values measuring the colocalization of all replication origins in our datasets with a series of publicly available chromatin modifications (see an example for AMI values in Additional file 1: Table S4). Since chromatin modification data were not available for our iPS and EB cells, we performed origin comparisons in those cells with chromatin modification data from h1ES and K562 cells, respectively. AMI values were standardized, clustered and represented as heat maps using the CIMminer tool [49]. Clustered replication origin associations are shown in Fig. 5 with strong associations depicted in deep red and weak associations depicted in blue. These analyses revealed that overall, a large majority of replication origins analyzed (in both cancer and non-cancer cells) were preferentially associated with a similar set of chromatin modifications including H3K4me3, H3K9Ac and unmethylated CpG islands. DNase hypersensitivity, RNA polymerase II binding sites, methylated CpG islands and H3K9me3 exhibited weaker colocalizations with origins (Fig. 5).

**Fig. 6:**
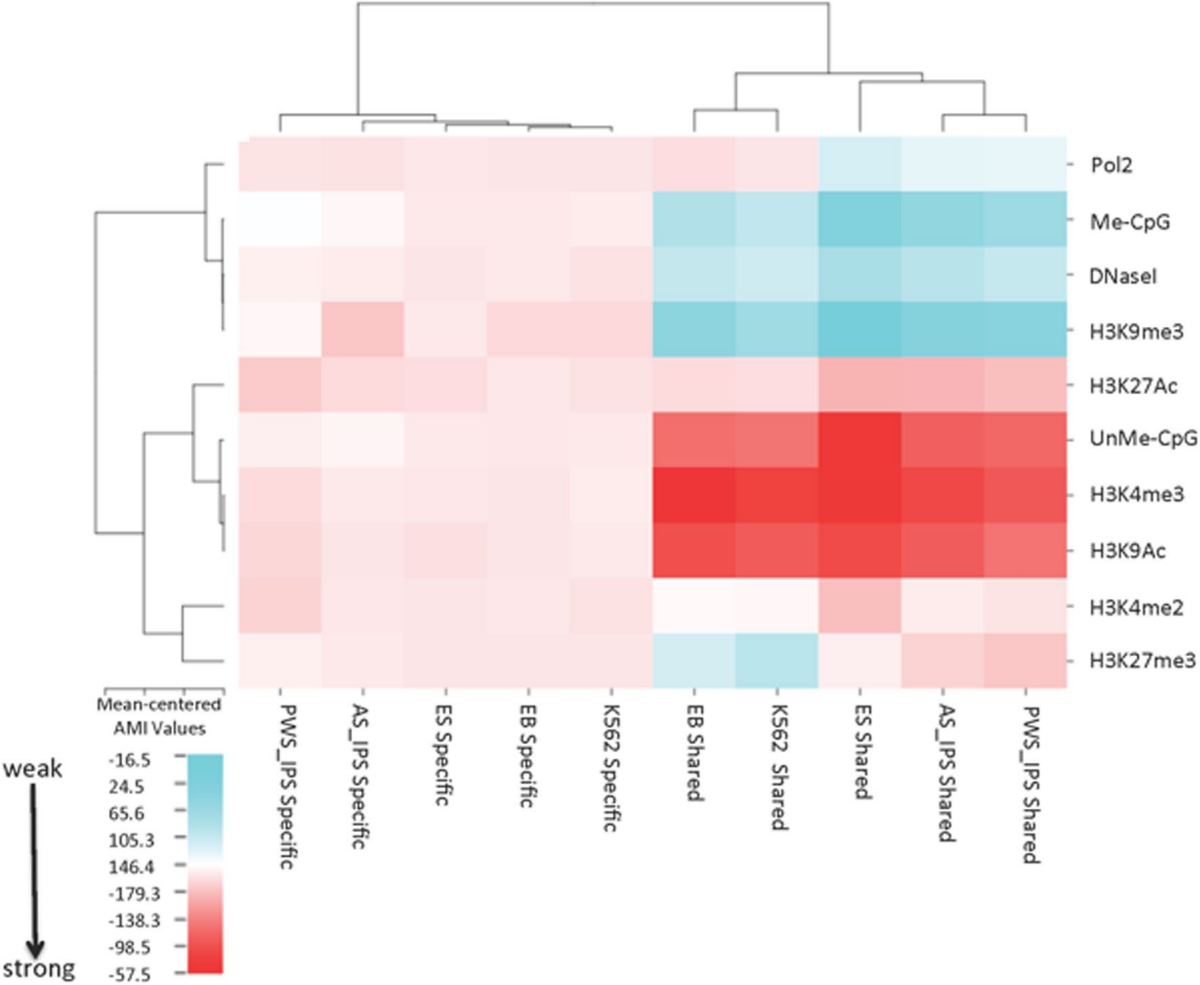
Shared and cell-type-specific replication origins clustered by association with chromatin features. Alignment of origins with chromatin modifications was performed using ColoWeb [48] as exemplified in Fig. 4. Heat maps representing the extent of preferential association of origins with distinct chromatin modifications were clustered by chromatin modifications and cell lines. The extent of association between origins and each modification is *color coded*, with deeper *red color* representing higher mean-centered AMI values and blue representing lower values. Shared and cell-type-specific replication origins clustered separately and displayed distinct associations with chromatin modifications

We next asked whether the association of histone modifications with replication origins was similar for shared and cell-type-specific origins. Shared origins associated strongly with the euchromatin markers H3K4me3, H3K9Ac and unmethylated CpG islands (Fig. 6) and weakly with methylated CpG islands, H3K9me3 and DNase hypersensitivity. Cell-type-specific origins exhibited stronger colocalization with the heterochromatin marker, H3K9me3, when compared with shared origins (Fig. 6). Cell-type-specific origins exhibited intermediate levels of colocalization with all other chromatin modifications analyzed. H3K27Ac exhibited similar colocalization with cell-type-specific and shared origins. Shared and cell-type-specific origins from EB and K562 cells (both of the myeloid lineage) clustered together, suggesting that replication origins from cells of the same lineage exhibit similar patterns.

### Shared and cell-type-specific origins associate with distinct regulatory domains

We used an independent approach to investigating whether replication origins are enriched in particular chromatin domains. Semiautomatic genome annotation (SAGE) partitions the genome into five distinct regulatory domains by incorporating histone modifications with measures of chromatin conformation [56]. This approach identifies three types of repressive domains and two types of active domains. Repressive domains include constitutive heterochromatin (CON), characterized by H3K9me3 and gene scarcity; facultative heterochromatin (FAC), characterized by H3K27me3 and a lack of gene expression; and quiescent domains (QUI), which are not characterized by any chromatin feature included in the algorithm. Facultative heterochromatin is thought to suppress gene activity in a tissue-specific manner, whereas quiescent domains are regions depleted of genes that occur in closed chromatin compartments. The two active domains include broad expression domains (BRD), characterized by transcription-associated chromatin markers including H3K36me3, and specific expression domains (SPC), characterized by regulatory markers such as H3K27Ac, which contain a large fraction of genes expressed only in certain cell types.

**Fig. 7:**
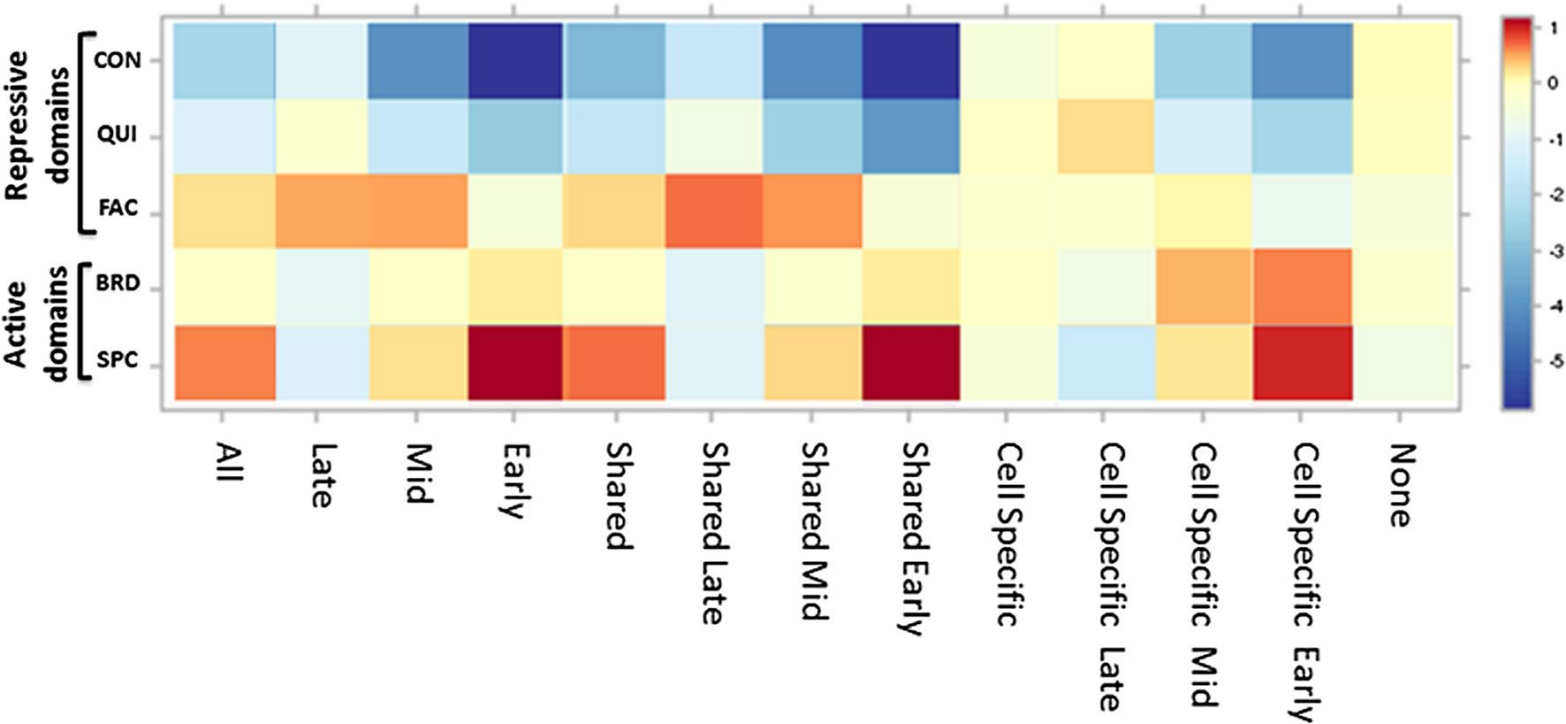
Association of subsets of EB replication origins with annotated genomic domains. Subsets of EB replication origins (all origins, shared origins and cell-type-specific origins) were stratified based on replication timing and investigated for their association with K562 genomic domains using SAGA analysis [56]. For each subgroup, the extent of enrichment for a particular domain is indicated on the scale of color bar. Repressive domains include constitutive heterochromatin (CON), facultative heterochromatin (FAC) and quiescent domains (QUI). Active domains include broad expression domains (BRD) and specific expression domains (SPC). The groups designated “early,” “late” and “middle” represent all origins stratified by replication time (during S-phase). The “none” group corresponds to all non-origin positions

Replication origins identified in the EB cells were divided into two sets: shared and cell-type-specific origins as well as origins replicating during early, middle and late S-phase (Fig. 7) [51]. Chromatin domains used for the SAGA analyses were identified in K562 cells, representing the erythroid lineage. In agreement with our colocalization analyses, SAGA found that all replication origins were enriched in SPC and depleted in CON and QUI domains. SAGA confirmed that shared replication origins were associated with active chromatin domains, whereas cell-type-specific origins showed no strong enrichment within any domain. Early replicating origins were enriched in SPC domains and were depleted from CON domains, whereas origins activated during middle and late S-phase both showed some enrichment in FAC domains.

### Shared and cell-type-specific origins are activated at distinct times during S-phase

**Fig. 8:**
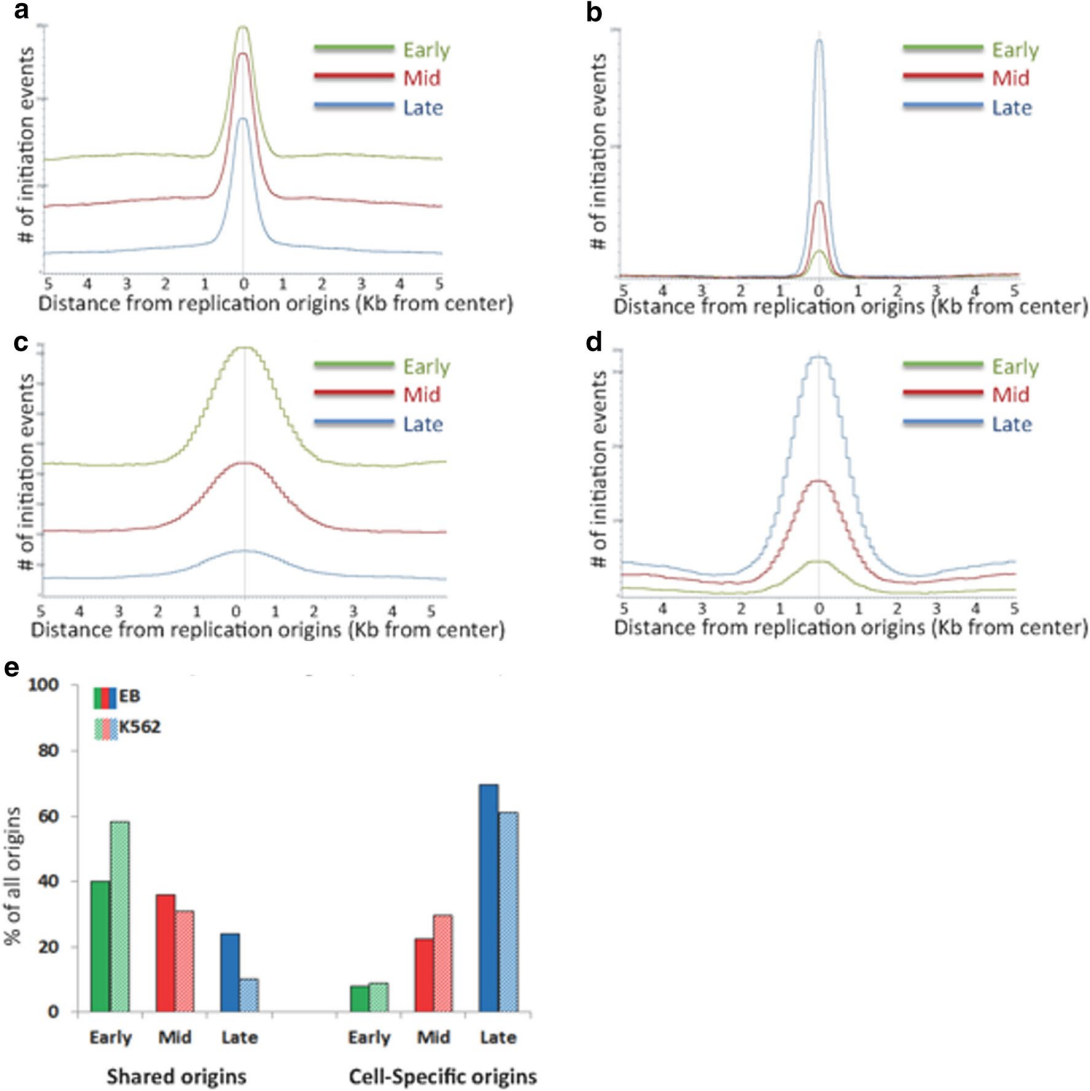
Timing of replication initiation in shared and cell-type-specific origins. Groups of **a** EB shared, **b** EB cell-type-specific, **c** K562 shared and **d** K562 cell-type-specific replication origins were stratified according to replication time. Replicating quintiles were created from BED files based on TimEX replication timing data for the EB cells [51] and Repli-seq for the K562 cell line [62]. The frequency of replication initiation in the first, third and fifth quintiles was plotted for genomic regions flanking replication origins. The histogram *x* axis extends 5-kb upstream and 5-kb downstream from the center of shared or cell-type-specific replication origins. The *y* axis represents the number of peaks shared among the indicated samples. Data are summarized in the histogram (**e**). *Bar graph* depicting the percent of shared (*left*) and cell-type-specific (*right*) origins found in each replication timing period. Shared replication origins exhibited a slight preference for early replication, whereas cell-type-specific replication origins were enriched in late timing stages

To determine whether shared and cell-type-specific replication origins were activated at distinct replication times, we separated the origins from EB cells into fractions (first, third and fifth quintiles—see “Methods” section for details) stratified by the timing of DNA replication initiation [51] and determined the proportion of shared or cell-type-specific origins at each time period (Additional file 1: Table S5; Fig. 8). Shared replication origins replicated preferentially in early and middle S-phase whereas cell-type-specific origins replicated most frequently in the late replicating fraction. For example, 46.2 % of EB cell-type-specific origins replicated during the final stage of S-phase (vs. 5.4 and 15 % for early and middle S-phase, respectively) (Fig. 8b, e; Additional file 1: Table S5). Similar results were obtained for K562 origins (Fig. 8c, d). These observations indicated that shared replication origins were not restricted temporally, whereas cell-type-specific origins preferentially replicated during late S-phase.

## Discussion

In this study, we characterized chromatin modifications associated with replication origins among several cell lines representing differentiated and undifferentiated states. We identified a shared set of origins used in all non-cancer and cancer cell lines tested, and groups of origins that are cell type specific. Cell lineage and differentiation status affected replication origin distribution, whereas cancer-specific origin profile variations were not observed. For both non-cancer and cancer cell lines, the shared set of origins was larger than the cell-type-specific set, and a large group of origins (about 50,000) initiated at identical locations in all cells. We observed a consistent epigenetic signature for shared and cell-type-specific replication origins across cell lines.

In all cell lines, we identified many more origin peaks than predicted from the 130–140 kb average inter-origin distance calculated using single fiber analyses in human cells [26, 57]. In concordance with previous studies [7, 9, 10, 54], we observed distances of ~10–30 kb between replication origin peaks. This apparent discrepancy reflects, at least in part, flexible origin choice, since in metazoans, many initiation sites are selected anew on each chromosome during every cell cycle. In addition, because origins can cluster within short distances, what appears as a single origin on a fiber can be seen as a cluster of reads in NS studies. Our observations provide strong support to models [2, 3, 28, 58], proposing that replication origins identified by population-based studies identify, in aggregate, all available initiation sites, with the frequency of site utilization reflecting factors such as chromatin structure, condensation and transcription.

**Fig. 9:**
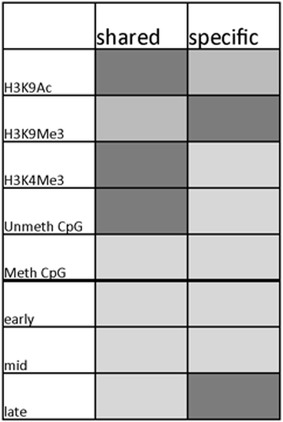
Summary of chromatin modifications associated with shared and cell-type-specific replication origins. Shared origins associated most strongly with unmethylated CpG islands, H3K4me3 and H3K9Ac, while cell-type-specific origins associated mostly with methylated CpG islands and H3K9me3, and preferentially replicated late

Shared origins (those utilized by all cell lines tested) exhibited a consistent epigenetic signature, most similar among cells from similar lineages (Fig. 9). These shared origins were enriched for CpG islands, in agreement with previous studies [7, 14, 54, 59]. CpG islands associated with shared origins, but not with cell-type-specific origins, were preferentially unmethylated. CpG islands were present in only ~10–20 % of replication origins, suggesting that association with unmethylated CpGs is not the sole factor in replication origin selection. Shared origins also showed distinct preferences for open chromatin markers (e.g., H3K4me3 and H3K9Ac) and were not enriched for methylated CpG islands, H3K9me3 or DNase-hypersensitive regions. These shared origins might be the hypothesized “master” origins, delineating origins that can be found in multiple cell lines of various differentiation states [60, 61].

Our analyses did not detect strong colocalization between DNase hypersensitivity and replication origins. This observation seems to differ from previous studies from our laboratory and others, which reported replication origin enrichment in DNase-hypersensitive regions [9, 10] and implicated DNase hypersensitivity in replication timing [58, 62]. In addition, a recent computational model [27] showed that cell-type-specific replication timing could be recapitulated in a cell line-specific manner if replication origins near DNase-hypersensitive sites initiated preferentially. However, the present study does not contradict the previous findings, because the current analyses were designed to detect chromatin features that associate preferentially with origins and not with adjacent sequences, whereas previous analyses measured overall rates of association. Together, the combined studies suggest that replication initiation events tend to occur in the vicinity of DNase-sensitive regions, but the precise locations of initiation events within those regions do not center on DNase-sensitive sites. The local determinants for replication origin utilization are likely based on the distinct transcriptional program or nuclear architecture [2, 28, 29, 63] characteristic of each individual cell line [9, 43]. Our analyses also suggest that cell-type specific replication origins that are used more frequently in the final stages of S-phase may be selected because of their proximity to DNase-hypersensitive sites.

Trimethylated histone H3 lysine (H3K9me3) preferentially associated with cell-type-specific replication origins, but not shared origins. In agreement, cell-type-specific origins preferentially initiated replication during late S-phase, consistent with the previously reported association of late replication origins within heterochromatin [30]. However, cell-type-specific origins exhibited lower, although still significant, associations with other chromatin modifications, including many of the open chromatin markers more strongly associated with shared origins. Hence, the association of H3K9me3 with cell-type-specific, but not shared origins, could indicate that H3K9 methylation facilitates initiation. Still, additional chromatin markers likely play roles in the choice of cell-type-specific origins. Notably, the H3K9me3 modification and one of its binding partners, HP1, interact with cellular machinery that primes chromatin for replication initiation [2, 64]. The ORC-associated protein ORCA interacts with H3K9 [65], and H3K9 methylation plays a role in the maintenance of large-scale constitutive and pericentric heterochromatin domains [66].

The observations reported here suggest that while shared origins exhibit similar local chromatin marks, cell-type-specific origins are less homogenous and can be divided into subgroups that might react differently to specific chromatin modifications. For example, while some cell-type-specific origins may represent a unique group associated with H3K9me3, another group may initiate replication in all cells, but exhibit signals below the detection threshold in some cell types, as previously described [15]. Thus, these origins may have a low association with active chromatin markers. Overall, our findings support the hypothesis that separate classes of replication origins respond differently to internal and external cues and can be chosen in a flexible manner that reflects cell-type-specific nuclear organization.

Our observations suggest that cellular differentiation affects replication initiation site location. For example, both shared and cell-type-specific K562 cell origins were most similar to origins from EB cells derived from the same erythroid lineage. Similarly, all pluripotent cell line origins exhibited similar epigenetic patterns, associating with acetylated and trimethylated H3K27 to a larger extent than origins in differentiated cell lines. These observations suggest that shared replication origins associate with H3K27 trimethylation at “bivalent promoters,” a hallmark of epigenetic plasticity in pluripotent cells [67, 68]. We also observed that EB replication initiation sites colocalized with H3K4me1 (data not shown), a histone modification that has been observed at promoters and enhancers of regions developmentally regulated during human erythropoiesis [69]. Data for H3K4me1 chromatin-binding sites from other cell lines are not available, prohibiting direct assessment of whether the association we observed also pertains to other cells. Taken together, these observations are consistent with the hypothesis that differentiation states affect origin selection patterns.

Replication origins can initiate replication ectopically regardless of differentiation status [34–38]. These observations suggest that origin activity can be determined, at least in part, by the primary sequence. In line with this, we found most replication origins to be shared, possibly contributing to the establishment of a decondensed chromosomal environment through associations with “open chromatin” modifications. Indeed, origins used to prevent transgene silencing and stabilize transcriptional activity in the context of gene expression vectors belong to the shared group [3, 5, 6]. In contrast, we observed that cell-type-specific origins colocalize with a different group of chromatin modifications, which may modulate origin activity in a differentiation-responsive manner. Combined with recent whole-genome analyses that identified sequence features common to many, but not all origins [11, 16, 26, 28, 63], our observations support the hypothesis that replication origins represent a diverse group of sequences that interact dynamically with the local chromosomal environment to establish a chromatin context that is permissive, but not obligatory, for DNA replication initiation. DNA sequences, therefore, appear to dictate the potential to initiate replication, whereas differentiation-associated changes in chromatin structure and modifications affect the decisions leading to activation of specific origins.

## Conclusions

Analyses of replication initiation patterns in human cells identified two distinct sets of replication origins, each exhibiting a consistent epigenetic signature. Shared replication origins were used in all cell lines tested, whereas cell-type-specific origins were consistently used in particular cells. Cancer-specific variations in origin profiles were not observed, whereas groups of origins from similar lineages and differentiation states exhibited high concordance. The shared set of origins was larger than the cell-type-specific set, and a large group of origins (about 40,000) initiated replication at identical locations in all cells. Shared origins replicated at all stages of S-phase and were enriched for unmethylated CpG islands and histone modifications typically associated with open chromatin. Cell-type-specific origins typically replicated late in S-phase and were associated with trimethylated histone H3 on lysine 9. Neither origin group exhibited a strong local preference for DNase-hypersensitive regions. Combined with previous studies demonstrating a role for DNA sequence in facilitating DNA replication initiation, our observations suggest that chromatin modifications and cellular differentiation control origin selection from a series of genetically predetermined potential initiation sites.

## Abbreviations

ORC: origin recognition complex; MCM: mini chromosome maintenance complex; CMG: CDC45, MCM10, GINS complex; Pre-RC: pre-replication complex; CGI: CpG island; NS-seq: nascent strand sequencing; IDR: irreproducible discovery rate; ROI: region of interest; AMI: above mean integral; SAGA: semi-automated genomes annotation algorithm; CON: constitutive heterochromatin; FAC: faculties heterochromatin; QUI: quiescent domains; SPC: specific expression domains; BRD: broad expression domains.

### Cell lines

K562: erythroleukemia cell line
MCF7: breast cancer cell line
HCT116: colorectal caner cell line
U2OS: osteosarcoma cell line
H1ES: human stem cell line
AS_iPS and PWS_iPS: iPS cell lines
EB: basophilic erythroblasts

## Additional file

10.1186/s13072-016-0067-3 Supplemental figures and tables.
