# Supplementary material for: Distinct epigenetic features of differentiation-regulated replication origins
Source: Epigenetics Chromatin. 2016 May 10;9:18. doi: 10.1186/s13072-016-0067-3 (PMC4862150; doi:10.1186/s13072-016-0067-3)

Supplemental Table Legends

Table S1a Replication origin profiles

Replication origins were identified by massively parallel sequencing of nascent strand preparations from the indicated cell lines isolated either by BrdU immunoprecipitation (BrdU) or by λ-exonuclease digestion (Exo). If both methods were used to map origins in a particular cell line, an asterisk indicates the mapping method used to generate data in the current paper. The number of peaks, average peak size, distance between the peaks and % of genome coverage are indicated. Citations in the rightmost (ref.) column refer to the sources of origin data (reference number if published and "this work" if unpublished) and citations below the table refer to the source of cell lines.

Table S1b Replicate comparisons. Comparisons among replicates of nascent strand preparations (HCT116, U2OS, K562) and among nascent strand peaks called against various preparations of genomic DNA. Five pairs of replicates were compared. The first pair contained peaks from two independent preparations of HCT116 nascent strands prepared using the λ-exonuclease method. The second pair contained peaks from HCT116 nascent strands isolated using the BrdU method and called against BrdU substituted and unsubstituted genomic DNA. The third pair contained peaks from two independent nascent strand preparations from U2OS cells. The fourth pair contained two independent preparations of K562 nascent strands (one prepared in this study and the other prepared independently in another lab with the resulting peaks accessed in GEO. The fifth pair contained peaks from a preparation of MCF7 nascent strands called

vs. G0 genomic DNA and λ-exonuclease digested genomic DNA. For each pair, "all peaks" represent the number of peaks in the specific replicate, "# of peaks colocalized with replicate peaks" represent the number of peaks in the replicate that were also present within 2 kb of peaks in the second replicate.

Table S1c Genomic distribution of replication origins.

Table S2a Characterization of shared and cell-specific replication origins in non-cancer cells. Groups of shared and cell-type specific origins identified among non-cancer cells are listed. BED files containing replication origin peaks were generated by the SICER program and used in sequential intersections (see Methods for details). Each row depicts the results of intersection between two BED files: the first containing origin peaks from the reference cell and the second containing origin peaks from one or more "comparison cell"(s). (Colocalized peaks were defined as peaks located within less than 2 kb of each other). For example, in row 1, "All Peaks" depicts the number of peaks in AS_iPS nascent strands (reference cell file); "Colocalized Peaks" depicts the number of peaks present in AS_iPS and PWS_iPS; "Specific Peaks" depicts the number of peaks present in AS_iPS and absent from the PWS_iPS file. Rows 2 and 3 follow the same patterns with the same reference cell file and with files containing origin peaks from ES and EB, respectively, as comparison cells. In row 4, the reference file is AS_iPS and the comparator file is a file containing peaks from both ES and EB. Hence, "All Peaks" depicts the number of peaks in AS_iPS nascent strands (reference cell file); "Colocalized

Peaks” depicts the number of peaks present in AS_iPS, ES and EB files; “Specific Peaks” depicts the number of peaks present in AS_iPS and absent from both ES and EB.

Table S2b Characterization of replication origins in cancer cells

Groups of shared and cell-type specific origins identified among cancer cells are listed using the same methodology and conventions described in the legend to Table S2A.

Table S3. Input and output for Chi squared test of independence for data shown in Table 2.

Table S4. Colocalization of replication origins with select chromatin marks. (AMI values correspond to the images shown in Fig. 4.)

Table S5. Distribution of EB and K562 replication origins during early, middle and late S-phase. (Values correspond to the images shown in Figure 8.)

Supplemental Figure Legends

Fig. S1 Screenshots of integrated genome viewer depicting replication initiation profiles in the (**a)** DHFR locus of chromosome 5, (**b)** HPRT locus of chromosome X and (**c)** LMNB2 locus of chromosome 19. For each screenshot, a chromosome map is shown in the top row with the mapped region delineated by a red rectangle. The second row shows the scale of the region being mapped and chromosomal coordinates are shown below the scale. RefSeq genes are shown underneath the chromosomal coordinates, above a

series of experimental tracks showing the distribution of replication origins in the nascent strand preparations from cell lines indicated on the left.

Fig. S2 Results of the Irreproducibility Discovery Rate (IDR) test comparing shared and cell-type specific origins. **a** Origin files from the two iPS cell lines (AS_IPS and PWS_iPS). **b** Origin files from AS_iPS and U2OS cells

Fig. S3 ColoWeb alignment of representative chromatin features with nascent strand abundance from several cancer and non-cancer cell lines. Graphic representations are shown as described in the Fig. 3 legend. Chromatin features depicted in the figure were also profiled in the graphs shown in Fig. 4

Table S1A

Name	Type of Cell	Cell Line Reference	# of origin peaks	Method	average peak length (bp)	average distance (kb) between peaks	% of genome covered	Ref.
EB	Basophillic Erythroblasts	Mukhopadyay et al, 2014	144,753	BrdU* + Exo	3809.3	16.9	18	51
ES	H1ES, Embryonic stem cell	Thomson et al, 1998	90,621	BrdU	4759.6	28.3	14	52
AS_IPS	iPS	Chamberlain et al, 2010	119,258	Exo	3793.9	21.3	15	This work
PWS_IPS	iPS	Chamberlain et al, 2010	124,707	Exp	3241.8	18.9	15	This work
K562	Erythroleukemia	Lozziio CB and Lozzio BB, 1975	201,952	BrdU + Exo*	3453.9	11.1	23	9 + This work
MCF7	Breast adenocarcinoma	Soule HD et al, 1973	161,358	Exo	3350.5	11.9	22	9
HCT116	Colorectal carcinoma	Brattain MG, et al, 1981	78,339	BrdU + Exo*	3861.6	34.3	10	50 + This work
U2OS	Osteosarcoma	Ponten J and Saksela E, 1967	92,210	Exo	3948.3	28.5	12	This work

Cell Line References:

EB: Mukhopadhyay R, Lajugie J, Fourel N, Selzer A, Schizas M, Bartholdy B, Mar J, Lin CM, Martin MM, Ryan M *et al*: **Allele-Specific Genome-wide Profiling in Human Primary Erythroblasts Reveal Replication Program Organization**. *PLoS genetics* 2014, **10**(5):e1004319.

ES: James A. Thomson, Joseph Itskovitz-Eldor, Sander S. Shapiro, Michelle A. Waknitz, Jennifer J. Swiergiel, Vivienne S. Marshall, and Jeffrey M. Jones: **Embryonic Stem Cell Lines Derived from Human Blastocysts** *Science 6 November 1998: 282 (5391), 1145-1147.*

iPS: Chamberlain SJ, Chen PF, Ng KY, Bourgois-Rocha F, Lemtiri-Chlieh F, Levine ES, Lalande M: **Induced pluripotent stem cell models of the genomic imprinting disorders Angelman and Prader-Willi syndromes**. *Proceedings of the National Academy of Sciences of the United States of America* 2010, **107**(41):17668-17673.

K562: Brattain MG, et al. Heterogeneity of malignant cells from a human colonic carcinoma. Cancer Res. 41: 1751-1756, 1981.

MCF7: Ponten J, Saksela E. Two established in vitro cell lines from human mesenchymal tumours. Int. J. Cancer 2: 434-447, 1967.

HCT116: Lozzio CB, Lozzio BB. Human chronic myelogenous leukemia cell-line with positive Philadelphia chromosome. Blood 45: 321-334, 1975.

U2OS: Soule HD, et al. A human cell line from a pleural effusion derived from a breast carcinoma. J. Natl. Cancer Inst. 51: 1409-1416, 1973.

* Both BrdU and lambda exonuclease based NS samples were sequenced with similar profiles; the results in the paper are based on a single preparation as indicated by * .

Table S1b

	# of all peaks	# of peaks colocalized with replicate peaks	% colocalized peaks
**HCT116 (Exo) replicate 1**	**78339**	**68023**	**87.1**
**HCT116 (Exo) replicate 2**	**109125**	**72870**	**66.8**
**HCT116 (BrdU) called vs. BrdU substituted genomic**	**65544**	**63296**	**96.5**
**HCT116 (BrdU) called vs. unsubstituted genomic**	**62573**	**62491**	**99.9**
**U2OS replicate 1**	**89300**	**65088**	**73.0**
**U2OS replicate 2**	**92210**	**62374**	**67.3**
**K562, GSE46189 (Picard et al)**	**37074**	**31335**	**84.5**
**K562, this study**	**201952**	**28334**	**16.0**
**MCF7 (Exo) called vs. G0 genomic**	**192461**	**173153**	**90.0**
**MCF7 (Exo) called vs. Exo digested genomic**	**161358**	**161358**	**100.0**

Table S1c

		% peaks in exons	% peaks in introns	% partially in exon/intron	% peaks in promoters	% intergenic peaks
AS_IPS	All	9.5	46.6	3.5	8.3	40.4
PWS_IPS	All	10.3	47.4	4	9.2	38.2
EB	All	8	46.3	4.1	7.4	41.6
ES	All	7.1	46	2.5	5.6	44.4
K562	All	7.7	48	1.2	5.2	43.2
MCF7	All	1	29.5	27.4	19.4	42
HCT116	All	12.6	46.4	5.8	12.8	35.2
U2OS	All	1	25.8	37	28.1	36.1
AS_IPS	Shared	1.0	23.9	42.7	34.1	32.4
	Cell-specific	1.1	37.6	8.1	5.7	53.2
PWS_IPS	Shared	1.0	23.8	43.1	33.7	32.2
	Cell-specific	2.2	42.5	10.2	7.0	45.2
ES	Shared	0.7	20.3	48.5	37.8	30.6
	Cell-specific	0.6	35.3	15.1	7.6	49.0
EB	Shared	1.0	23.4	43.3	34.3	32.4
	Cell-specific	1.1	38.9	10.8	6.8	49.2
HCT116	Shared	1.1	22.9	44.5	34.6	31.6
	Cell-specific	2.3	26.8	31.8	27.6	39.1
MCF7	Shared	0.8	20.9	46.7	35.9	31.6
	Cell-specific	1.0	36.6	12.5	7.2	49.9
U2OS	Shared	1.0	22.5	45.6	35.4	30.9
	Cell-specific	1.2	32.4	13.4	8.5	53.0
K562	Shared	0.8	21.7	45.8	35.4	31.7
	Cell-specific	1.1	37.5	14.1	8.0	47.3

Table S2a

Row Number	Reference Cell	Comparison Cell	Peaks in reference cells	Colocalized Peaks (in both reference and comparison cells)	Specific Peaks (only in reference)	% Colocalized	% Specific
1	AS_IPS	PWS_IPS	119258	104887	15056	87.9	12.6
2	AS_IPS	ES	119258	70431	48964	59.1	41.1
3	AS_IPS	EB	119258	102045	17898	85.6	15.0
4	AS_IPS	ES and EB	119258	67431	14896	56.5	12.5
5	PWS_IPS	AS_IPS	124707	110105	25292	88.3	20.3
6	PWS_IPS	ES	124707	80130	54734	64.3	43.9
7	PWS_IPS	EB	124707	114905	20492	92.1	16.4
8	PWS_IPS	ES and EB	124707	68893	16471	55.2	13.2
9	ES	AS_IPS	90621	67264	23357	74.2	25.8
10	ES	PWS_IPS	90621	71770	18851	79.2	20.8
11	ES	EB	90621	78119	12502	86.2	13.8
12	ES	EB and AS_IPS	90621	64921	10159	71.6	11.2
13	ES	EB, AS_IPS, and PWS_IPS	90621	62477	8507	68.9	9.4
14	EB	AS_IPS	144753	102127	42625	70.6	29.4
15	EB	PWS_IPS	144753	110019	34733	76.0	24.0
16	EB	ES	144753	85920	58832	59.4	40.6
17	EB	ES and AS_IPS	144753	69106	25811	47.7	17.8
18	EB	ES, AS_IPS and PWS_IPS	144753	65466	18366	45.2	12.7

Table S2b

Row Number	Reference Cell	Comparison Cell	Peaks in reference cells	Colocalized Peaks (in both reference and comparison cells)	Specific Peaks (only in reference)	% Colocalized	% Specific
1	K562	MCF7	201952	135194	41880	66.9	20.7
2	K562	HCT116	201952	80002	122651	39.6	60.7
3	K562	U2OS	201952	91206	111447	45.2	55.2
4	K562	MCF7 and HCT116	201952	76994	38872	38.1	19.2
5	K562	MCF7, HCT116, and U2OS	201952	64972	35266	32.2	17.5
6	MCF7	K562	192451	130245	32368	67.7	16.8
7	MCF7	HCT116	192451	78407	114711	40.7	59.6
8	MCF7	U2OS	192451	88347	104771	45.9	54.4
9	MCF7	K562 and HCT116	192451	74932	28893	38.9	15.0
10	MCF7	K562, HCT116, and U2OS	192451	62930	26100	32.7	13.6
11	HCT116	K562	78339	68143	7773	87.0	9.9
12	HCT116	MCF7	78339	71419	7440	91.2	9.5
13	HCT116	U2OS	78339	64881	13978	82.8	17.8
14	HCT116	K562 and MCF7	78339	68539	4893	87.5	6.2
15	HCT116	K562, MCF7, and U2OS	78339	57873	1319	73.9	1.7
16	U2OS	K562	92210	77973	11281	84.6	12.2
17	U2OS	MCF7	92210	80495	12318	87.3	13.4
18	U2OS	HCT116	92210	67628	25185	73.3	27.3
19	U2OS	K562 and MCF7	92210	76784	7570	83.3	8.2
20	U2OS	K562, MCF7, and HCT116	92210	60631	3955	65.8	4.3

Table S3: Input and Output for Chi squared test of Independence

A: AS_IPS input

AS_IPS	ORI-CGI	ORI not-CGI	row sum
Shared	16998	47451	64449
Specific	948	14841	15789
Partial	4038	31449	35487
column sum	21984	93741	115725

B: PWS_IPS input

PWS_IPS	ORI-CGI	ORI not-CGI	row sum
Shared	17272	51621	68893
Specific	799	16182	16981
Partial	4382	40970	45352
column sum	22453	108773	131226

C: ES input

ES	ORI-CGI	ORI not-CGI	row sum
Shared	16935	39751	56686
Specific	76	15270	15346
Partial	1125	17464	18589
column sum	18136	72485	90621

D: EB input

EB	ORI-CGI	ORI not-CGI	row sum
Shared	32749	91738	124487
Specific	69	3924	3993
Partial	4460	31387	35847
column sum	37278	127049	164327

E: Normal Cell Output

	AS_IPS	PWS_IPS	ES	EB
df	2	2	2	2
Chi sq value	5350.01	6694.59	9359.48	4075.59
p-value	<0.0001	<0.0001	<0.0001	<0.0001

F: K562 input

K562	ORI-CGI	ORI not-CGI	row sum
Shared	10824	37968	48792
Specific	549	37385	37934
Partial	8532	88328	96860
column sum	19905	163681	183586

G: MCF7 input

MCF7	ORI-CGI	ORI not-CGI	row sum
Shared	8228	35227	43455
Specific	278	25727	26005
Partial	2400	82222	84622
column sum	10906	143176	154082

H: HCT116 input

HCT116	ORI-CGI	ORI not-CGI	row sum
Shared	9565	36930	46495
Specific	387	1002	1389
Partial	9007	19049	28056
column sum	18959	56981	75940

I: U2OS input

U2OS	ORI-CGI	ORI not-CGI	row sum
Shared	10522	37198	47720
Specific	122	31985	32107
Partial	7194	29417	36611
column sum	17838	98600	116438

J: Cancer Cell Output

	K562	MCF7	HCT116	U2OS
df	2	2	2	2
Chi sq value	10370.8	13030.75	1248.41	7719.08
p-value	<0.0001	<0.0001	<.0001	<.0001

Table S4:

Modification	MCF7	K562	ES	EB
H3K27Ac	41.3	40.8	138.9	57.4
H3K4Me3	57.9	36.7	109.5	53.9
UnMe-CpG	68.3	40.3	188.4	91.4
Me-CpG	15.5	9.3	27.3	25.6
Pol2	37.4	36.4	85.6	62.8
H3K9Me3	26.5	15.1	84.7	15.7
DNAse1	21.8	22.2	61.9	27.9

Table S5

Reference file	Comparator file	Reference peaks #	Comparator peaks #	Ref. peaks colocalized with comparator
EB shared	EB early quintile	122,603	32,714	30,392 (40.1%)
EB shared	EB mid quintile	122,603	32,715	27,275 (36%)
EB shared	EB late quintile	122,603	32,713	18,092 (23.9%)
				Total peaks: 75,758 (100%)
EB specific	EB early quintile	3,950	32,714	207 (7.9%)
EB specific	EB mid quintile	3,950	32,715	591 (22.5%)
EB specific	EB late quintile	3,950	32,713	1,825 (69.6%)
				Total peaks: 2,623 (100.000%)
K562 shared	K562 early quintile	64,972	11,546	8,486 (58.5%)
K562 shared	K562 mid quintile	64,972	11,545	4,507 (31.1%)
K562 shared	K562 late quintile	64,972	11,545	1,514 (10.4%)
				Total peaks: 14,507 (100%)
K562 specific	K562 early quintile	35,266	11,546	489 (9.2%)
K562 specific	K562 mid quintile	35,266	11,545	1,585 (29.2%)
K562 specific	K562 late quintile	35,266	11,545	3,263 (61.1%)
				Total peaks: 5,337 (100%)

Figure S1A

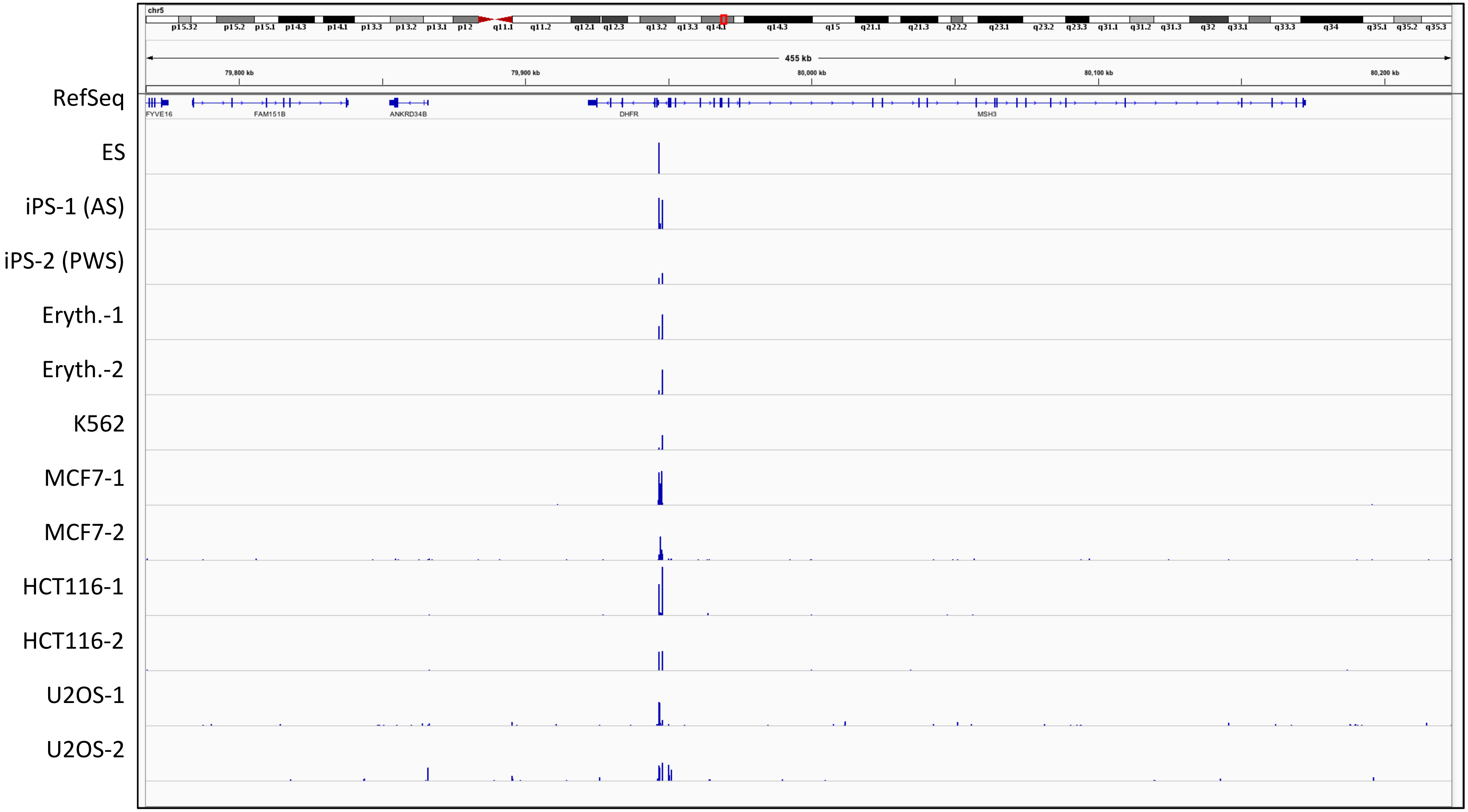
chr5
455 kb
79,800 kb
79,900 kb
80,000 kb
80,100 kb
80,200 kb
RefSeq
FYVE16
FAM151B
ANKRD34B
DHFR
MSH3
ES
iPS-1 (AS)
iPS-2 (PWS)
Eryth.-1
Eryth.-2
K562
MCF7-1
MCF7-2
HCT116-1
HCT116-2
U2OS-1
U2OS-2

Figure S1B

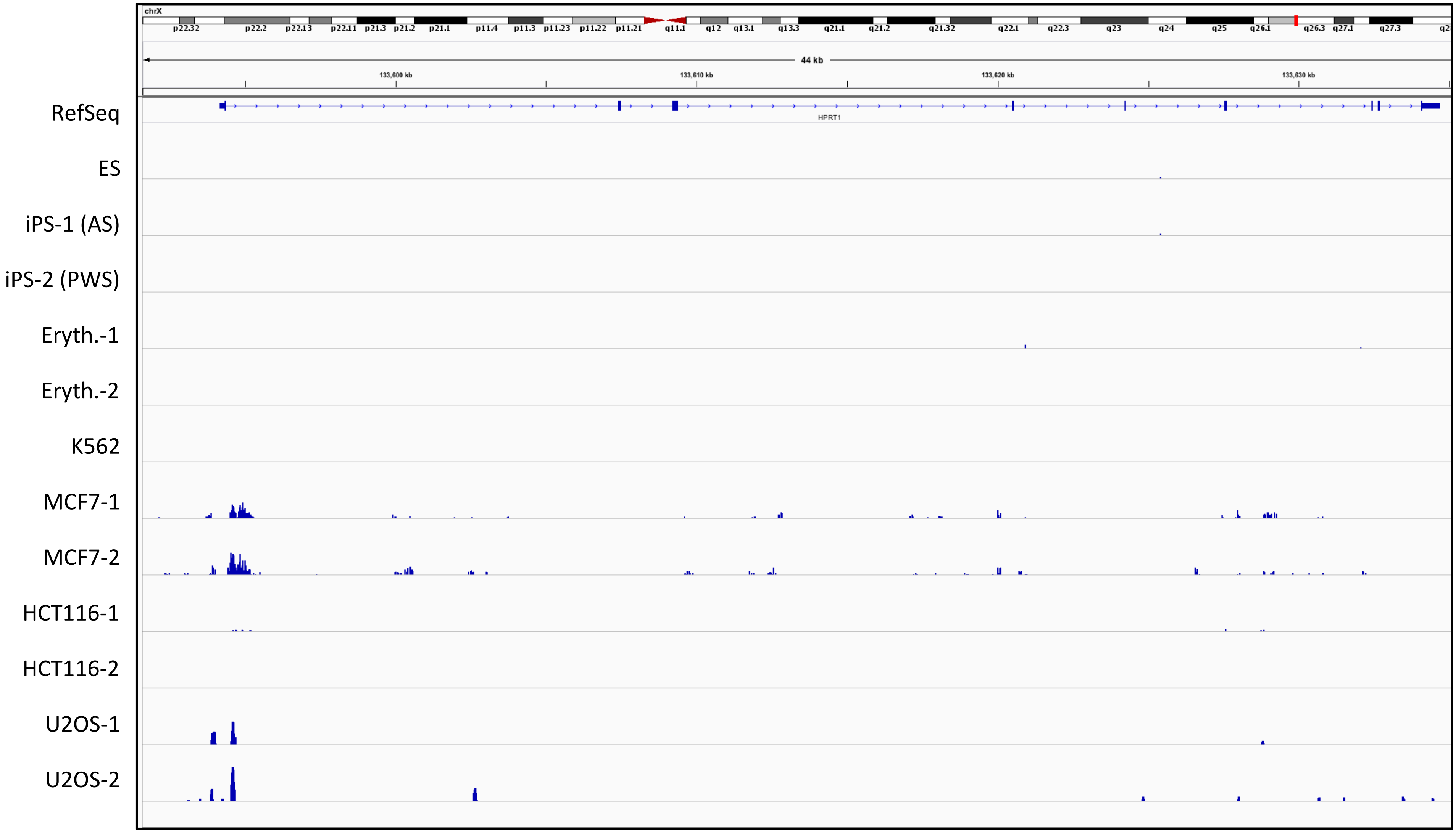
chrX
p22.32
p22.2
p22.13
p22.11
p21.3
p21.2
p21.1
p11.4
p11.3
p11.23
p11.22
p11.21
q11.1
q12
q13.1
q13.3
q21.1
q21.2
q21.32
q22.1
q22.3
q23
q24
q25
q26.1
q26.3
q27.1
q27.3
44 kb
133,600 kb
133,610 kb
133,620 kb
133,630 kb
RefSeq
HPRT1
ES
iPS-1 (AS)
iPS-2 (PWS)
Eryth.-1
Eryth.-2
K562
MCF7-1
MCF7-2
HCT116-1
HCT116-2
U2OS-1
U2OS-2

Figure S1C

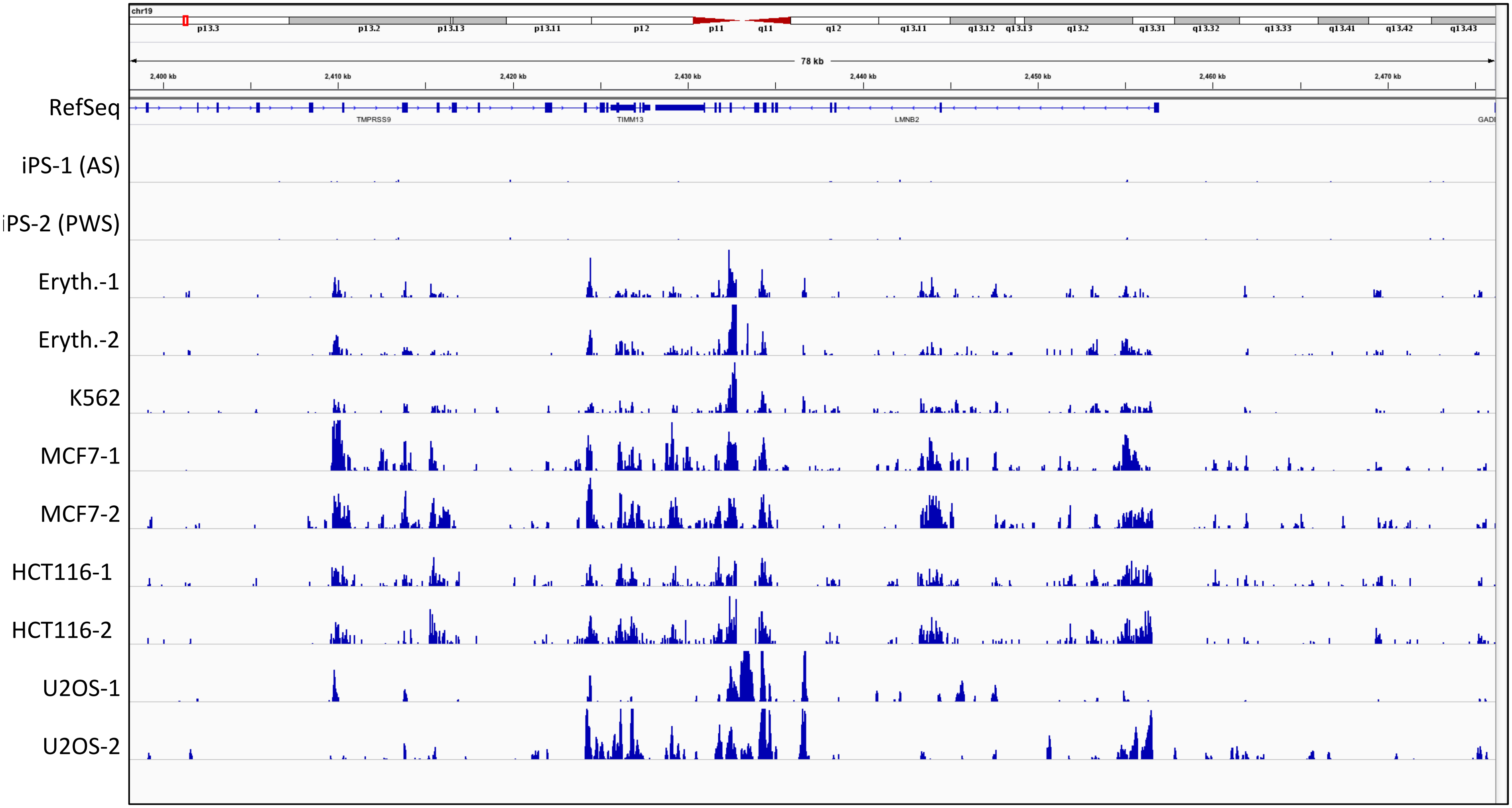
chr19
p13.3
p13.2
p13.13
p13.11
p12
p11
q11
q12
q13.11
q13.12
q13.13
q13.2
q13.31
q13.32
q13.33
q13.41
q13.42
q13.43
78 kb
2,400 kb
2,410 kb
2,420 kb
2,430 kb
2,440 kb
2,450 kb
2,460 kb
2,470 kb
RefSeq
TMPRSS9
TIMM13
LMNB2
GADI
iPS-1 (AS)
iPS-2 (PWS)
Eryth.-1
Eryth.-2
K562
MCF7-1
MCF7-2
HCT116-1
HCT116-2
U2OS-1
U2OS-2

Figure S2: Comparison of reproducibility using Irreproducibility Discovery Rate (IDR)

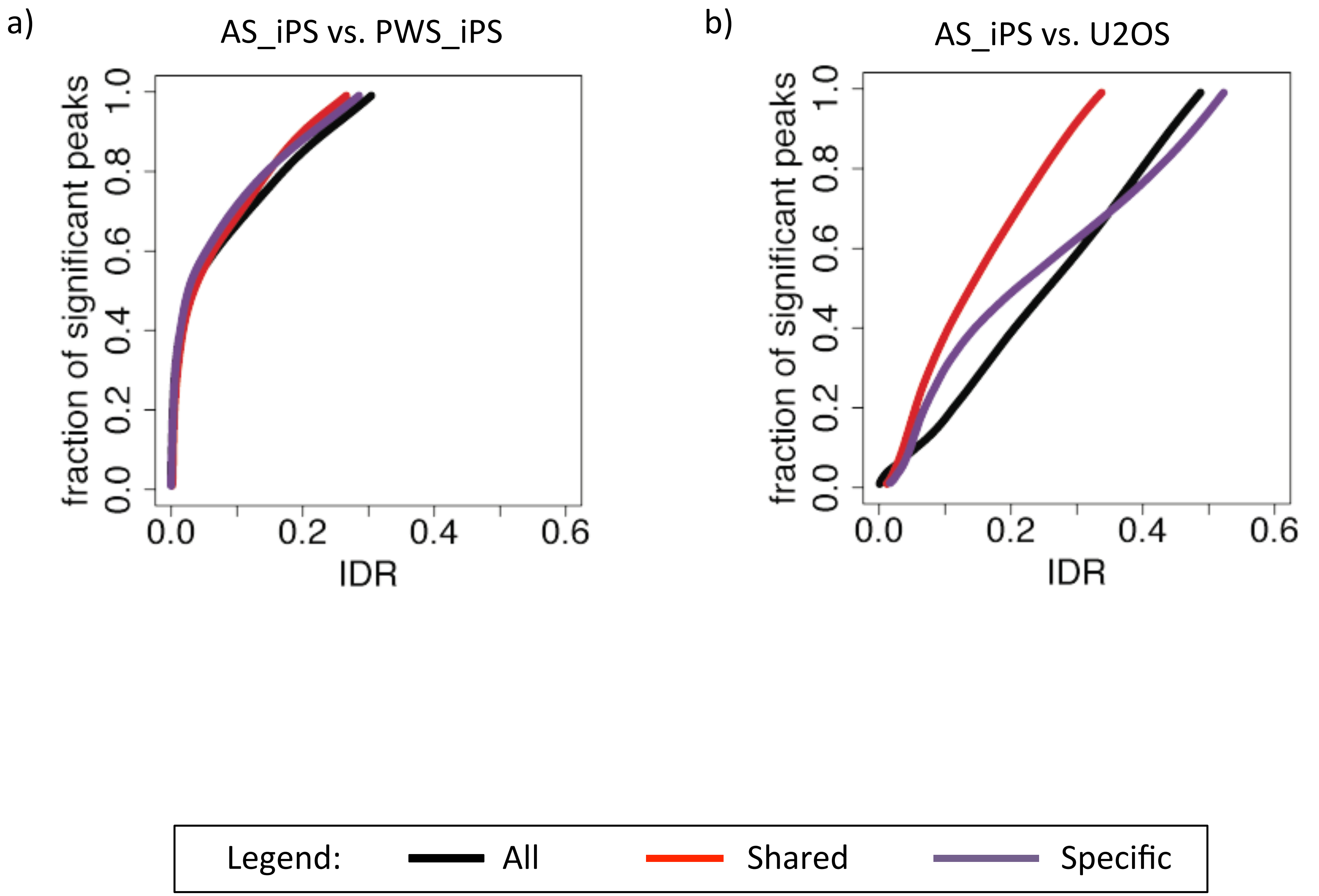

Figure S3

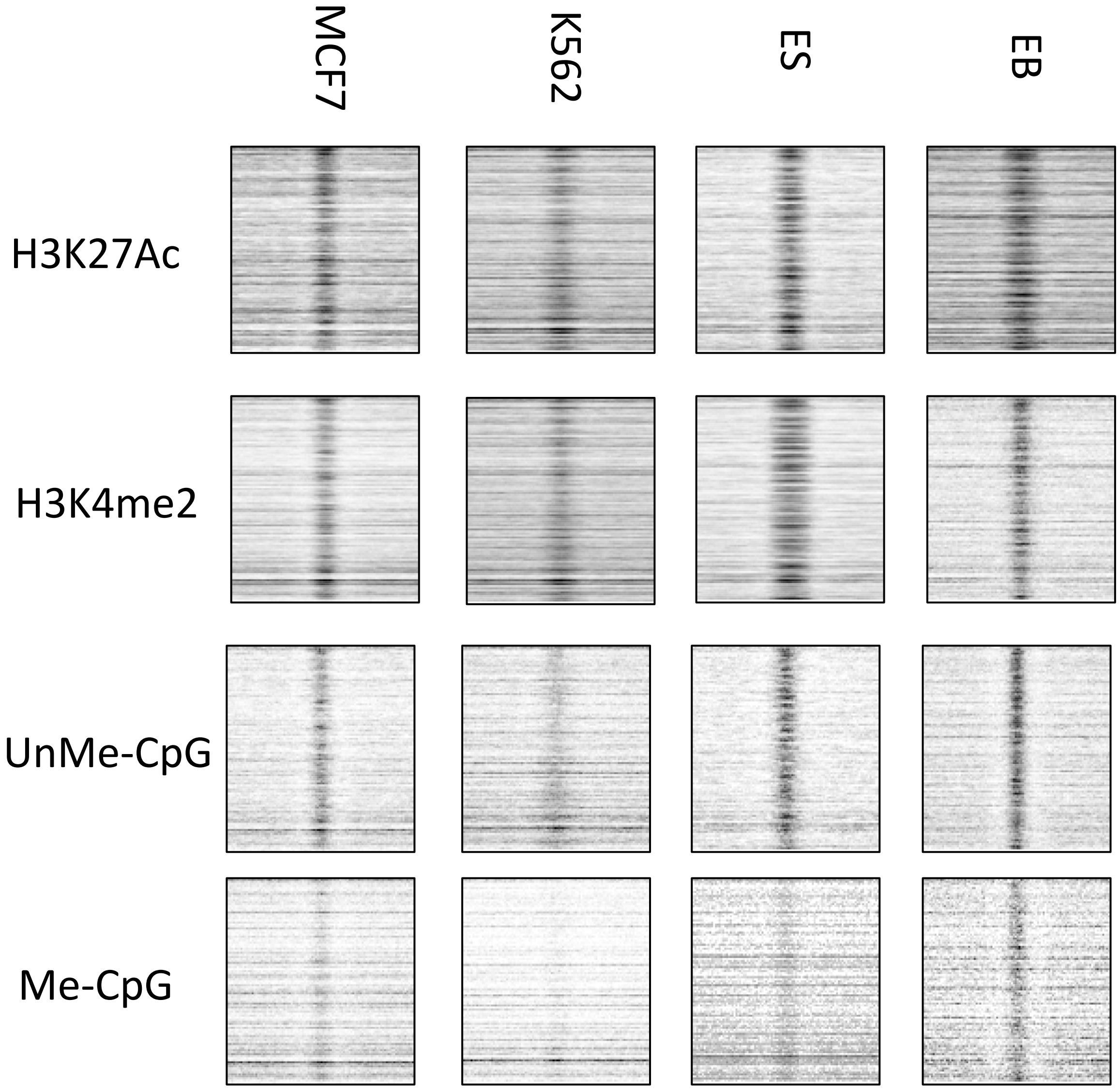

Supplement: Supplementary file 1 — 10.1186/s13072-016-0067-3 Supplemental figures and tables. [file 13072_2016_67_MOESM1_ESM.pdf]
